# Collaboration, impact, and research trends at the veterinary research institutes of the Chinese Academy of Agricultural Sciences: a bibliometric analysis from 2009 to 2023

**DOI:** 10.1186/s12917-026-05531-7

**Published:** 2026-05-16

**Authors:** Zaib Ur Rehman, Muhammad Jahandad Basra, Adnan Hassan Tahir, Zahid Manzoor, Shanhui Ren, Alia Afzal, Chunchun Meng

**Affiliations:** 1 Shanghai Veterinary Research Institute (SHVRI), Chinese Academy of Agricultural Sciences (CAAS), Shanghai, 200241 P. R. China; 2 Institute of Animal Sciences, PMAS Arid Agriculture University, Rawalpindi, 46300 Pakistan; 3 Faculty of Veterinary and Animal Sciences, PMAS Arid Agriculture University, Rawalpindi, 46300 Pakistan; 4 Lanzhou Veterinary Research Institute (LVRI), Chinese Academy of Agricultural Sciences (CAAS), Lanzhou, 730030 PR China; 5 Faculty of Social Sciences, PMAS Arid Agriculture University, Rawalpindi, 46300 Pakistan

**Keywords:** Veterinary Research Institutes, Chinese Academy of Agricultural Sciences, Bibliometric analysis, VOSviewer, Science mapping

## Abstract

This paper examines the research output and impact of Veterinary Research Institutes (VRIs) of the Chinese Academy of Agricultural Sciences (CAAS), namely Shanghai Veterinary Research Institute (SHVRI), Harbin Veterinary Research Institute (HVRI), and Lanzhou Veterinary Research Institute (LVRI), to answer research questions. The study explores publication count, citation structure, h-index, most-cited articles, keyword trends, trend topics, co-authorship, thematic maps, and bibliographic coupling. The data is extracted from Scopus, covering publications from 2009 to 2023. Utilizing Excel, R (Biblioshiny), and VOSviewer (version 1.6.20, Leiden University, Netherlands), we found that VRIs of the CAAS demonstrated a steady upward trend in their research output, with SHVRI leading in top-cited articles, HVRI in citations and h-index, and LVRI in publications. Key journals include Veterinary Microbiology and Parasites and Vectors. Leading authors are Chan Ding (SHVRI), Xiaomei Wang (HVRI), and Xing-Quan Zhu (LVRI). Key research focus is on parasitology, virology, immunology, and molecular biology. SHVRI and HVRI’s demonstrated focus is on virology, while LVRI’s interest is in genetic analyses of the pathogens. Bibliographic coupling highlights core themes: SHVRI on Newcastle disease, *Schistosoma japonicum*, *Toxoplasma gondii*, and Porcine reproductive and respiratory syndrome virus; HVRI on African swine fever virus, Influenza viruses, and Porcine epidemic diarrhea virus; and LVRI on *Toxoplasma gondii*, the parasitic mitochondrial genome, and Foot and Mouth disease virus. Findings reflect CAAS’s commitment to addressing critical animal diseases for China’s development and food security.

**Supplementary Information:**

The online version contains supplementary material available at 10.1186/s12917-026-05531-7.

## Introduction

Global veterinary research plays a crucial role in the “One Health” framework, serving as a frontline defence against zoonotic diseases, contributing to global food security, and advancing the development of new vaccines and therapeutics [1–4]. There has been a rise in emerging infectious diseases worldwide; consequently, research in veterinary science has been promoted by national agricultural research systems to counteract the economic and public health threats [5, 6]. In this context, PR China is seen as a major contributor in addressing these threats, with the Chinese Academy of Agricultural Sciences (CAAS) as one of the key players whose mission is dedicated to agricultural innovation and animal disease control [4]. In the field of veterinary sciences, CAAS ranks second in research percentile according to the Scimago Institutions Rankings, demonstrating its position as a contributor and an efficient model for evaluating institutional growth [7].

The CAAS has 3 veterinary research institutes (VRIs), which are strategically located in different provinces, namely, Shanghai Veterinary Research Institute (SHVRI), Harbin Veterinary Research Institute (HVRI), and Lanzhou Veterinary Research Institute (LVRI). These institutes have research groups working on various diseases and animals, in line with the CAAS’s directions and priorities. Among the contributions, research aimed at the development of diagnostic capabilities [8–10], epidemiological surveillance [11, 12], the relationships between hosts and pathogens [13–19], and vaccines [20–26] against different diseases.

The current study used bibliometric analysis tools to gain a comprehensive understanding of the scientific contributions of the VRIs of the CAAS. Bibliometric analysis employs statistical methods to identify research and citation trends and to unpack the thematic structure of a specific topic. It helps in examining collaborative networks, research themes, and evaluating the evolution of research prospects by utilizing scholarly outputs over time [27]. It identifies gaps, provides novel ideas for innovation, and gives a one-stop overview of a subject matter [28]. Similar retrospective studies are common for different topics related to animal and human diseases [1, 29–33].

Bibliometric analysis has enabled policymakers to translate institutional reputation into strategic and actionable insights, especially in the case of research institutions for evidence-based evaluation [34, 35]. While national-level and discipline-wise research in veterinary and agricultural sciences is substantial, a granular comparative assessment of individual institutes under an umbrella institute remains undiscovered in the literature. This gap is more evident in the case of the Chinese Academy of Agricultural Sciences (CAAS), along with its constituent veterinary research institutes, namely SHVRI, HVRI, and LVRI, which have never been the subject of a systematic and longitudinal bibliometric study covering their 15-year scientific evolution (2009–2023). This is particularly a decisive time frame because it includes the devastating outbreak of African Swine Fever in PR China in August 2018 [36] and the disruptive outbreak of the SARS-CoV-2 pandemic, both of which are known to have entirely redefined research priorities in veterinary research. Furthermore, the social structure of scientific collaboration in these institutes, which DJ Price and DD Beaver [37] refer to as the “Invisible College,” and the co-authorship network that undergirds knowledge production has not been mapped or measured [38, 39]. By using performance analysis and bibliographic coupling to perform thematic mapping simultaneously [40], the given study not only outlines the specialized research portfolios of each institute but also determines a crucial gap in the research of emerging subjects, which include antimicrobial resistance in zoonotic pathogens, a WHO-designated global health priority [41, 42], which has yet to be prioritized as a research focus by the VRIs of the CAAS. This observation outlines a clear path to follow for future funding and global cooperation. Therefore, this study aims to answer the following research questions (RQs) to conduct an evidence-based evaluation of VRIs of the CAAS from 2009 to 2023.

- RQ1: How has the scientific productivity and citation impact changed over time in the three VRIs of CAAS between 2009 and 2023, and what does this tell us about their institutional growth trajectory?
- RQ2: What are the high-impact publications that establish the international scientific presence of VRIs, and what are the core pathogens or fields that they focus on?
- RQ3: Who are the key academic personalities that influence the research output in these institutes, and how is their scientific reputation in the form of h, g, and m indices compared in the three VRIs?
- RQ4: Which intellectual descriptors (author keywords) do the basic research pillars of each institute describe?
- RQ5: How did the research themes change with time in response to emerging biological threats?
- RQ6: How are the co-authorship networks of these VRIs organized, and how do these collaborative social networks contribute towards the production of knowledge throughout the CAAS?
- RQ7: What conceptual themes and research directions emerge from the publications of VRIs?
- RQ8: What core thematic clusters and specialized research focuses are revealed through the bibliographic coupling of publications from each VRI

These research questions delve into the academic development of CAAS and its VRIs. Research Question 1 breaks down trends in publication output and citation impact over time, thus plotting the growth curves of each institute. Research Question 2 focuses on high-impact articles and key pathogens that support the international scientific reputation of the VRIs. To measure intellectual leadership, Research Question 3 examines the impact of the leading scholars by comparing their reputation with the h, g, and m indices. Research Question 4 investigates the author keywords that define the fundamental research pillars of each institute. Furthermore, Research Question 5 traces the changing thematic focus over the years concerning emerging biological threats, using a trend topic analysis of publication titles. To capture the social structure of the academy, Research Question 6 maps the co-authorship network structure and its contribution to the creation of collective knowledge in the CAAS. Lastly, Research Question 7 deconstructs the thematic concepts and research paths that are enacted in structural thematic maps of abstracts within articles, and Research Question 8 outlines fundamental thematic clusters and specialized research areas with the help of bibliographic coupling.

## Materials and methods

### Data collection

The bibliometric data for the present study were retrieved on September 7, 2024. The data were collected from Scopus, a widely recognized database of peer-reviewed literature and citation information. The current study focused on three major veterinary research institutes in China, all under the Chinese Academy of Agricultural Sciences (CAAS): the Shanghai Veterinary Research Institute (SHVRI), the Harbin Veterinary Research Institute (HVRI), and the Lanzhou Veterinary Research Institute (LVRI). The search strings used to collect the data were:

- AFFIL ( “Shanghai Veterinary Research Institute” ) AND PUBYEAR > 2008 AND PUBYEAR < 2024 AND ( LIMIT-TO ( LANGUAGE, “English” ) ) AND ( LIMIT-TO ( DOCTYPE, “le” ) OR LIMIT-TO ( DOCTYPE, “re” ) OR LIMIT-TO ( DOCTYPE, “ar” ) ) AND ( LIMIT-TO ( SRCTYPE, “j” ) )
- AFFIL ( “Harbin Veterinary Research Institute” ) AND PUBYEAR > 2008 AND PUBYEAR < 2024 AND ( LIMIT-TO ( LANGUAGE, “English” ) ) AND ( LIMIT-TO ( DOCTYPE, “le” ) OR LIMIT-TO ( DOCTYPE, “re” ) OR LIMIT-TO ( DOCTYPE, “ar” ) ) AND ( LIMIT-TO ( SRCTYPE, “j” ) )
- AFFIL ( “Lanzhou Veterinary Research Institute” ) AND PUBYEAR > 2008 AND PUBYEAR < 2024 AND ( LIMIT-TO ( LANGUAGE, “English” ) ) AND ( LIMIT-TO ( DOCTYPE, “le” ) OR LIMIT-TO ( DOCTYPE, “re” ) OR LIMIT-TO ( DOCTYPE, “ar” ) ) AND ( LIMIT-TO ( SRCTYPE, “j” ) )

The data was restricted to publications between January 1, 2009, and December 31, 2023. The dataset included bibliographic metadata, citation counts, abstracts, keywords, and author affiliation details. The impact of the contributors was assessed using h, g, and m indices.

### Data analysis

Data analysis was conducted using a suite of tools. These included Microsoft Excel, R (biblioshiny package), and VOSviewer (version 1.6.20, Leiden University, Netherlands). To clean, manage, and tabulate data, Excel was used. The biblioshiny package in R was used to create analyses, including word clouds, trend topics, and thematic maps. VOSviewer (version 1.6.20, Leiden University, the Netherlands) was used to develop scientific landscapes, including co-authorship and bibliographic coupling analysis. The scientific maps are illustrated with coloured thematic nodes, where the size of each node represents the number of co-occurrences. The strength of the nodes is demonstrated by the spatial proximity and thickness of the connecting lines, with close proximity and thick lines representing a high degree of co-occurrence. We addressed an ambiguity caused by author names due to spelling and formatting in VOSviewer (version 1.6.20, Leiden University, Netherlands), where authors with similar names were not distinguished properly. To resolve this issue, Scopus author ID(s) were used for all author-related metrics. For the Biblioshiny packages and VOSviewer analyses, a customized synonym-reducing file was employed to reduce term redundancy. This customized file was created by first exporting an Excel file for each analysis from Biblioshiny, and a text file from VOSviewer containing the data illustrated in the figures and in tabular format. This data was adjusted in such a way that each recurring word was counted only once to reduce unwanted synonyms of a single word across the figures.

## Results

### Corpus of publications

Table 1 shows the publication and citation trends of the different VRIs of the CAAS (RQ1). The total number of documents retrieved was 6,010, comprising 1,474 publications from SHVRI, 2,176 from HVRI, and 2,360 from LVRI. SHVRI published a total of 1,474 documents between 2009 and 2023, averaging approximately 98.26 publications per year. These documents received a total of 29,881 citations, averaging 20.27 citations per article. SHVRI had its peak in 2022, with 170 publications. The year 2016 had the highest citation rate of 6,687; however, 2015 was the most influential year with an h-index of 27.

**Table 1:** Corpus of articles published by the veterinary research institutes of the Chinese Academy of Agricultural Sciences, 2009–2023

Year	Shanghai Veterinary Research Institute				Harbin Veterinary Research Institute				Lanzhou Veterinary Research Institute			
	NP	TC	C/*P*	H	NP	TC	C/*P*	H	NP	TC	C/*P*	H
2009	25	620	24.80	15	51	1983	38.88	24	18	566	31.44	12
2010	41	1052	25.66	20	81	2706	33.41	29	70	1610	23.00	22
2011	49	1937	39.53	22	102	2803	27.48	30	127	3247	25.57	34
2012	89	2429	27.29	26	107	3200	29.91	30	130	3522	27.09	33
2013	76	1729	22.75	26	146	4188	28.68	37	139	3759	27.04	32
2014	70	1412	20.17	22	133	3774	28.38	33	150	4135	27.57	31
2015	99	2701	27.28	27	161	3710	23.04	32	158	3919	24.80	32
2016	110	6687	60.79	26	155	3647	23.53	30	177	4067	22.98	35
2017	99	1678	16.95	23	174	3904	22.44	35	158	3814	24.14	31
2018	102	1815	17.79	25	147	3809	25.91	32	173	3650	21.10	30
2019	99	2355	23.79	23	159	3718	23.38	30	202	3532	17.49	29
2020	149	1818	12.20	20	199	6109	30.70	36	220	2741	12.46	24
2021	155	2000	12.90	22	162	2872	17.73	27	236	4167	17.66	25
2022	170	1191	7.01	16	229	2973	12.98	24	208	1563	7.51	19
2023	141	457	3.24	10	170	797	4.69	12	194	675	3.48	11

Here, *NP* Number of publications, *TC* Total citations, *h*  h index, *C/P *Citations per publications

Similarly, HVRI produced 2,176 publications during the same period, averaging 145.06 per year. These articles garnered 50,193 citations, averaging 123.06 per article. HVRI had its highest output in 2022, with 229 publications. In terms of citations, 2020 was the standout year, with 6,109 total citations; however, 2013 was the most influential year with an h-index of 37 (Table 1).

LVRI, as presented in Table 1, published 2,360 documents between 2009 and 2023, with an average of 157.33 publications per year. These documents received 44,967 total citations, with an average of 19.05 citations per article. The highest number of publications occurred in 2021, with 236 documents. In terms of citations, 2021 led with 4,167 total citations, while 2016 was the most influential year, achieving an h-index of 35. This suggests that LVRI produces more publications than SHVRI and HVRI, while HVRI produces higher-quality publications.

### Most cited articles

The top 20 cited documents published by the authors from SHVRI between 2009 and 2023 are presented in Table S1 (RQ2). It comprises 4 reviews, 1 letter, and 15 research articles. The highly cited document was a review, titled, “Guidelines for the use and interpretation of assays for monitoring autophagy (3rd edition)” published in 2016 in ‘Autophagy’. This review has been cited 4347 times. The next most cited document was also a review article published in 2019 in ‘Frontiers in Immunology’ entitled “The C/EBP homologous protein (CHOP) transcription factor functions in endoplasmic reticulum stress-induced apoptosis and microbial infection” [43]. This review has been cited 650 times. The third most cited document published by SHVRI was related to *Toxoplasma gondii* [44].

The top 20 most cited articles published by the authors of the HVRI between 2009 and 2023 are presented in Table S2. It contains 3 reviews, 1 letter, and 16 research articles (RQ2). The highly cited document was a research article titled “Susceptibility of ferrets, cats, dogs, and other domesticated animals to SARS-coronavirus 2”. This article was published in 2020 in ‘Science’ and have 1317 citations. The second highly cited document was a review article titled “Omicron variant of SARS-CoV-2: Genomics, transmissibility, and responses to current COVID-19 vaccines”. This review article was published in 2022 in the ‘Journal of Medical Virology’ and has been cited 555 times. The third highly cited document was a review article entitled “COVID-19: Epidemiology, Evolution, and Cross-Disciplinary Perspectives” [45]. This review article was published in 2020 in ‘Trends in Molecular Medicine’ and has been cited 424 times.

The top 20 cited articles published by the authors of the LVRI between 2009 and 2023 are presented in Table S3 (RQ2). These contain 6 reviews and 14 research articles. The highly cited document published by LVRI was a review published in 2021 in ‘Autophagy’ titled “Guidelines for the use and interpretation of assays for monitoring autophagy (4th edition)1”. This review article received 1494 citations. The second highly cited document was a research article entitled “The genomes of four tapeworm species reveal adaptations to parasitism”, published in 2013 in ‘Nature’. This article received 557 citations. The third highly cited document was a 2014 review in ‘The Lancet Infectious Diseases’ entitled “Severe fever with thrombocytopenia syndrome, an emerging tick-borne zoonosis”. This review article has been cited 405 times.

The comparison of the top 20 most-cited articles from 2009 to 2023 shows that SHVRI’s review article, “Guidelines for the use and interpretation of assays for monitoring autophagy (3rd edition)” is the highly cited document, with 4,347 TC, among the top documents from VRIs of CAAS. The most studied topics in the most cited articles are *Toxoplasma gondii*, *Schistosoma japonicum*, African Swine Fever, and Avian influenza. *Toxoplasma gondii* is an opportunistic protozoan that causes toxoplasmosis, a zoonotic disease [46]. Avian influenza and *Schistosoma japonicum* (snail fever) are of zoonotic importance [47, 48]. African swine fever is most studied as it severely threatens the pig industry of China [49].

### Journals publishing the research of VRIs of CAAS

The top 15 journals publishing articles of SHVRI are listed in Table 2. ‘Veterinary Microbiology’ is the leading journal with a number of publications (NP) 92, total citations (TC) 1836, and an h-index of 25. Journal of Virology comes next with NP 49, TC 1829, and an h-index of 25. Plos One remains third with NP 45, TC 1220, and an h-index of 20. The Journal of Virology is trending because it has only 49 publications, yet its h-index is equivalent to that of the top journal, and its total citations closely match those of Veterinary Microbiology.

**Table 2:** Top 15 journals by publication volume for veterinary research institutes of CAAS (2009–2023)

Sr. No.	Shanghai Veterinary Research Institute				Harbin Veterinary Research Institute				Lanzhou Veterinary Research Institute			
	Journal	NP	TC	H	Journal	NP	TC	H	Journal	NP	TC	h
1	VETERINARY MICROBIOLOGY	92	1836	25	VETERINARY MICROBIOLOGY	147	3356	33	PARASITES AND VECTORS	147	3877	32
2	JOURNAL OF VIROLOGY	49	1829	25	JOURNAL OF VIROLOGY	143	4485	41	PARASITOLOGY RESEARCH	94	1373	22
3	PLOS ONE	45	1220	20	ARCHIVES OF VIROLOGY	89	1443	22	INFECTION, GENETICS AND EVOLUTION	74	1536	25
4	FRONTIERS IN MICROBIOLOGY	44	501	13	VIRUSES	84	1098	19	FRONTIERS IN MICROBIOLOGY	65	1013	17
5	PARASITOLOGY RESEARCH	42	451	12	PLOS ONE	71	1896	25	VIROLOGY JOURNAL	63	1327	21
6	VIRUSES	42	450	12	FRONTIERS IN MICROBIOLOGY	70	887	17	JOURNAL OF VIROLOGY	61	1369	19
7	PARASITES AND VECTORS	40	647	13	VIROLOGY JOURNAL	60	1088	18	VETERINARY PARASITOLOGY	56	1211	22
8	VIROLOGY JOURNAL	34	676	15	JOURNAL OF VIROLOGICAL METHODS	57	972	19	PLOS ONE	50	1237	22
9	ARCHIVES OF VIROLOGY	34	639	14	VIRUS RESEARCH	50	862	18	EXPERIMENTAL PARASITOLOGY	43	707	17
10	VIRUS RESEARCH	30	506	14	TRANSBOUNDARY AND EMERGING DISEASES	47	1427	18	ACTA TROPICA	43	562	15
11	JOURNAL OF VIROLOGICAL METHODS	26	269	10	VACCINE	38	958	18	JOURNAL OF ANIMAL AND VETERINARY ADVANCES	36	133	7
12	VETERINARY PARASITOLOGY	26	424	12	VIRUS GENES	35	488	14	BIOMED RESEARCH INTERNATIONAL	35	510	13
13	SCIENTIFIC REPORTS	25	635	16	SCIENTIFIC REPORTS	33	861	20	FRONTIERS IN IMMUNOLOGY	32	855	16
14	VETERINARY RESEARCH	25	297	12	VIROLOGY	33	895	16	VETERINARY MICROBIOLOGY	32	569	15
15	FRONTIERS IN CELLULAR AND INFECTION MICROBIOLOGY	20	182	7	PLOS PATHOGENS	31	1508	18	ARCHIVES OF VIROLOGY	31	342	10

*VRIs* Veterinary Research Institutes, *CAAS* Chinese Academy of Agricultural Sciences, *NP* Number of publications, *TC* Total citations, *h* h index

The top 15 journals publishing the articles of HVRI are presented in Table 2. The maximum number of documents of HVRI are published in Veterinary Microbiology (147), while the most influential works are published in the Journal of Virology. The status of Veterinary Microbiology as one of the leading publication outlet for both SHVRI and HVRI (Table 2) reflects the institutes’ commitment to high-quality, field-specific peer-reviewed research within the international veterinary community.

As shown in Table 2, top journals publishing articles of LVRI are Parasite and Vector, Parasitology Research, Infection, Genetics, and Evolution, etc. Parasite and Vector have published 147 articles, with 3877 citations, and an h-index of 32. The second most productive journal is Parasitology Research, while the second most influential journal is Infection, Genetics, and Evolution. The comparison of the h-index for the top 15 journals publishing CAAS VRIs articles from 2009 to 2023 shows that the most influential journal is the Journal of Virology, with an h-index of 41 for HVRI publications, followed by Veterinary Microbiology, with an h-index of 33 for HVRI publications.

### Top author of the VRIs of the CAAS

The top 15 authors of SHVRI are listed in Table 3 (RQ3). Chan Ding is the top author with NP 250, TC 10,211, and an h-index of 38. Guangzhi Tong is the second most prolific author with NP 207, TC 4350, and an h-index of 35. Shengqing Yu and Xiangan Han are emerging influential authors with NP 151 and 105, and h-indexes of 30 and 25, respectively.

The most productive authors of HVRI are presented in Table 3 (RQ3). Xiaomei Wang is the most productive author with NP 184, while Hualan Chen is the most cited (9840), the most influential author with an h-index of 52, and the second most productive author with 179 NP.

**Table 3:** Top 15 authors having publications affiliated with veterinary research institutes of CAAS by publication volume (2009–2023)

Sr. No.	Shanghai Veterinary Research Institute				Harbin Veterinary Research Institute				Lanzhou Veterinary Research Institute			
	Author Name	NP	TC	H	Author Name	NP	TC	H	Author Name	NP	TC	h
1	Chan Ding	250	10,211	38	Xiaomei Wang	184	3232	31	Xing-Quan Zhu	549	14,584	52
2	Guangzhi Tong	207	4350	35	Hualan Chen	179	9840	52	Hong Yin	279	4234	30
3	Shengqing Yu	151	3613	30	Yulong Gao	178	3128	30	Jianxun Luo	240	3493	28
4	Zhi-Yong Ma	122	1815	25	Xiaole Qi	171	3007	30	Guiquan Guan	185	2500	26
5	Xiangan Han	105	1920	25	Zhigao Bu	158	8137	46	Xiangtao Liu	178	3804	35
6	Jiaojiao Lin	104	1621	23	Xuehui Cai	158	3297	34	Haixue Zheng	177	3264	33
7	Wu Tong	98	1531	20	Hua-Ji Qiu	140	4076	33	Dong-Hui Zhou	136	3195	32
8	Jian-Chao Wei	95	1384	20	Li Feng	134	3108	33	Xuepeng Cai	127	2328	22
9	Yafeng Qiu	95	1309	20	Changjun Liu	129	1937	24	Zhijie Liu	126	2334	26
10	Tongling Shan	92	1677	20	Hongyu Cui	120	2185	26	Huichen Guo	126	2969	31
11	Yanjun Zhou	88	1976	26	Siguo Liu	116	1662	22	Youquan Li	124	1851	23
12	Hai Yu	84	1353	23	Yanping Zhang	112	1686	23	Hany M. Elsheikha	106	2223	26
13	Ke Liu	83	984	19	Yongqiang Wang	111	2334	27	Junlong Liu	106	1505	22
14	Donghua Shao	82	1292	20	Li Gao	110	1671	22	Jifei Yang	105	1709	25
15	Beibei Li	82	1256	20	Kai Li	109	1577	22	Guangyuan Liu	100	1386	21

*VRIs* Veterinary Research Institutes, *CAAS* Chinese Academy of Agricultural Sciences, *NP* Number of publications, *TC* Total citations, *h* h index

The most prolific author of LVRI is Xing-Quan Zhu, with 549 NP, 14,584 TC, and an h-index of 52. Hong Yin is the second most productive (279) and cited (4234) author, while Xiangtao Liu is the second most influential author with an h-index of 35.

In summary, Xing-Quan Zhu of LVRI is the most influential author, with an h-index of 52 and 549 publications cited 14,584 times. Hualan Chen from HVRI is the most emerging influential author of VRIs in CAAS, with an h-index of 52 for just 179 publications.

### Word cloud of the most popular keywords

**Fig. 1:**
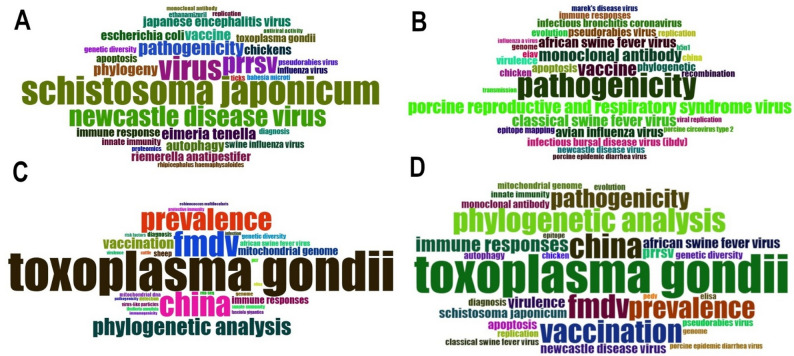
Word cloud visualization of author keywords (2009–2023) across the three institutions and CAAS overall. Maps were generated using Biblioshiny (R package) based on keywords that occurred at least 30 times. The font size and central placement of terms reflect their relative frequency and importance within the dataset. Panels represent: **A** SHVRI, **B** HVRI, **C** LVRI, and (**D**) CAAS

The word cloud is indicative of the frequently occurring terms, thus, the size of the term is representative of its importance in its topics [50]. The word cloud analysis of each VRI is conducted based on author keywords to answer the *RQ4* and it was performed using biblioshiny from R studio along with a synonym-reducing file, which was used to reduce the term redundancy. Based on author keywords, we set a threshold of 30 key terms. For SHVRI, the most significant terms include “*Schistosoma japonicum”* (87), “virus” (73), “newcastle disease virus” (62), “prrsv” (62), “pathogenicity” (44), and “*Eimeria tenella*” (36), respectively (Fig. 1-A). This suggests that *Schistosoma japonicum* is the frequently used keyword in publications from SHVRI or most of the publications from SHVRI are centered around this. Furthermore, the *Schistosoma japonicum* parasite is endemic to China, which explains its importance [48].

As shown in the Fig. 1-B, “pathogenicity” (110) as the most significant term, followed by “vaccine” (64), “porcine reproductive and respiratory syndrome virus” (62), “monoclonal antibody” (58), “classical swine fever virus” (53), and “african swine fever virus” (48), respectively.

The Fig. 1-C depicts “*Toxoplasma gondii*” (271) as the most dominantly occurring term, followed by “fmdv” (173), “china” (165), “prevalence” (151), “phylogenetic analysis” (117), and “vaccination” (78), respectively for the LVRI. Here the most dominant term is *Toxoplasma gondii*. It suggests that *Toxoplasma gondii* is the most occurred keyword might be due to higher publications of LVRI on this topic. It is most studied due to its zoonotic importance and its prevalence in humans in China [46].

The overall assessment of word cloud analysis of CAAS reveals “*Toxoplasma gondii*” (316) as the most substantial key term. The other keywords of relevant significance include “china” (203), “fmdv” (195), “phylogenetic analysis” (193), “vaccination” (188), “prevalence” (175), “pathogenicity” (155), and “immune responses” (124), respectively (Fig. 1-D).

### Trend topics

**Fig. 2:**
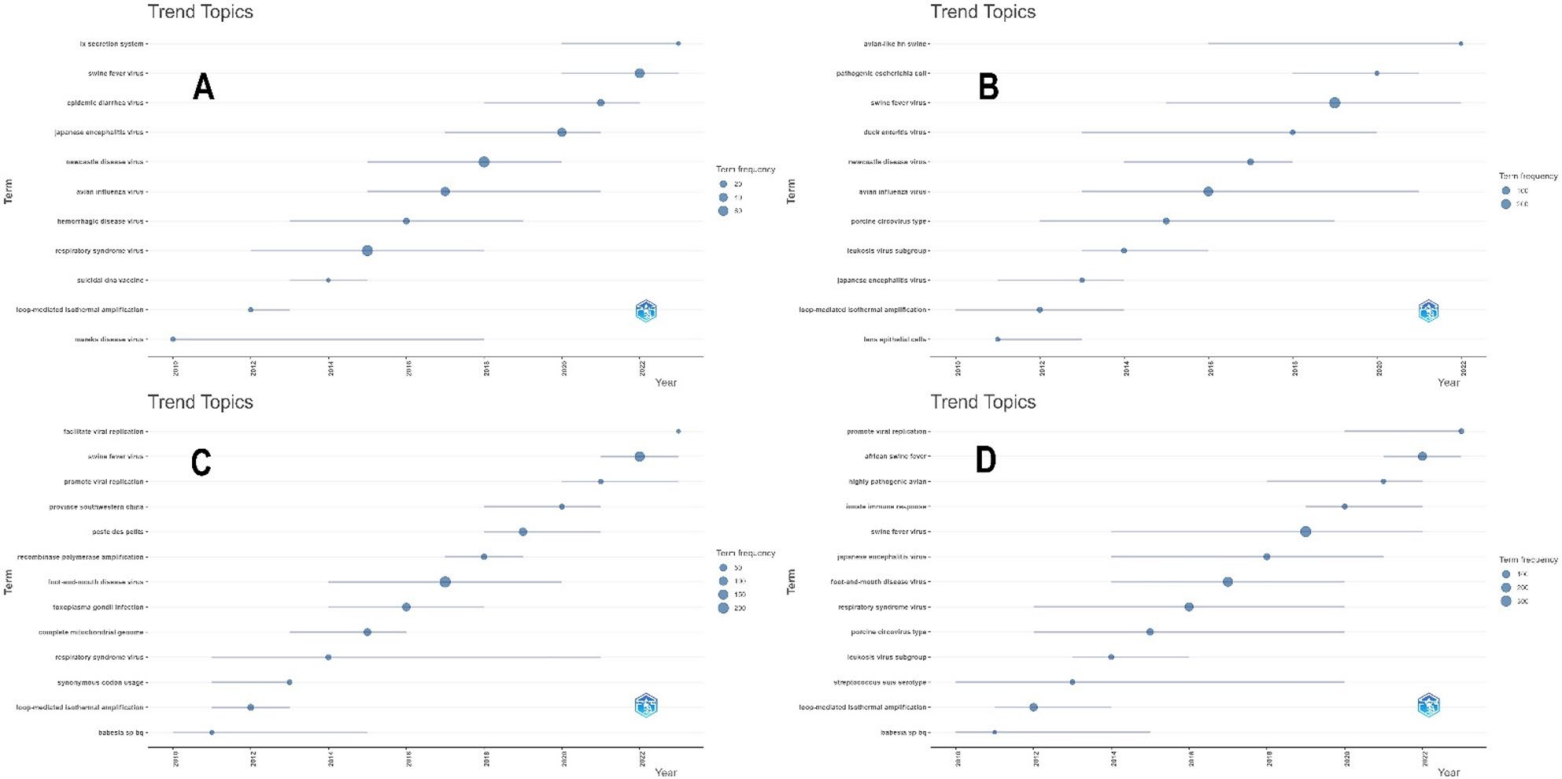
Evolution of trend topics based on article titles (2009–2023). Analysis was conducted using Biblioshiny (R package) with a minimum word frequency of 5 and a density of 1 word per year, with a synonym-reduction filter. Node size represents word frequency, while horizontal lines indicate the temporal duration of research focus for: **A** SHVRI, **B** HVRI, **C** LVRI, and (**D**) CAAS

The trend topics analysis shows the most researched topics over the years based on bibliographic data (Fig. 2). The horizontal axis shows the years during which the topics gained attention, while the vertical axis illustrates the trigrams (three consecutive words), that are the topics of concern. The trigrams are extracted from the titles of the articles to answer the *RQ5*. Trend topic analysis for all the VRIs was carried out based on a minimum word frequency of 5, 1 word per year, and a synonym-reducing file.

Figure 2-A shows the analysis for SHVRI based on titles with trigrams, which indicate 3 consecutive words and word stemming (the parameter was set to yes). These parameters were set for the recurring trend topics analysis of all the VRIs in this paper and the overall assessment of CAAS. From 2015 to 2020, the “newcastle disease virus” had the most frequent occurrence (78), indicating focused research on this virus. Around that period, the “respiratory syndrome virus” also steadily paced in its research area (75). This virus was studied mostly from 2012 to 2018. The most recently researched area of interest is the “swine fever virus” (53) currently peaking from 2020 to 2023 as this virus entered China in 2018 and spread rapidly [49].

In the case of HVRI, Fig. 2-B shows that the major trigram was the “swine fever virus” (289). This topic reached its peak from 2015 to 2022. Following it is the “avian influenza virus” (181), mostly searched from 2013 to 2021. Moreover, “newcastle disease virus” (46) was mostly researched from 2014 to 2018. The focus on viruses is mostly illustrated in this institute because the top 3 occurring trigrams are viral diseases.

The Fig. 2-C shows trend topics of the LVRI, where the “foot-and-mouth disease virus” (202) gained prominence from 2014 to 2020. After this, the “swine fever virus” (157) was researched from 2021 to 2023. Following it is “*Toxoplasma gondii* infection” (78) which was mostly searched from 2014 to 2018.

The overall assessment of CAAS is depicted in Fig. 2-D, which reveals that viruses are the primary focus of research. The “swine fever virus” (316) gained significant attention, especially from 2014 to 2022. Research on the “foot-and-mouth disease virus” (228) focused mostly on the period from 2014 to 2020. Similarly, the “african swine fever” (154) was mostly studied from 2021 to 2023. The “respiratory syndrome virus” (151) was a major research area from 2012 to 2020.

### Co-authorship of the authors

The co-authorship analysis shows the international/national collaborations among the authors, institutions, or countries. It was conducted using VOSviewer version 1.6.20. These are intellectual collaborations assessed using co-authorship analysis [51]. These collaborations create social networks [27], particularly international collaborations, which, when published in internationally renowned journals, contribute significantly to global scientific progress [52]. In this analysis, node size indicates the number of documents authored or co-authored by the individual. The link connecting two nodes shows the frequency of collaboration among the authors. Throughout the co-authorship analysis, some close collaborations are seen inside the clusters, forming a social network. Crane referred to it as “invisible college”, which shows that these social networks indicate close interactions between researchers, thereby influencing interesting prospects for further research [38]. The number of documents for some of the authors may differ from Table 3, as indicated above. The authors’ documents in this analysis are ambiguous regarding author specificity, due to VOSviewer’s inability to differentiate authors with similar names. However, Table 3 is counted using the unique author ID for an accurate measure. Consequently, the authors’ documents are recorded in Table 3. Although the ambiguity is minimal, as shown in Table 3, it ensures that the author’s collaboration network is accurate here. Therefore, the co-authorship analysis determines the collaborative patterns among the institutes’ affiliated authors to address *RQ6.*

**Fig. 3:**
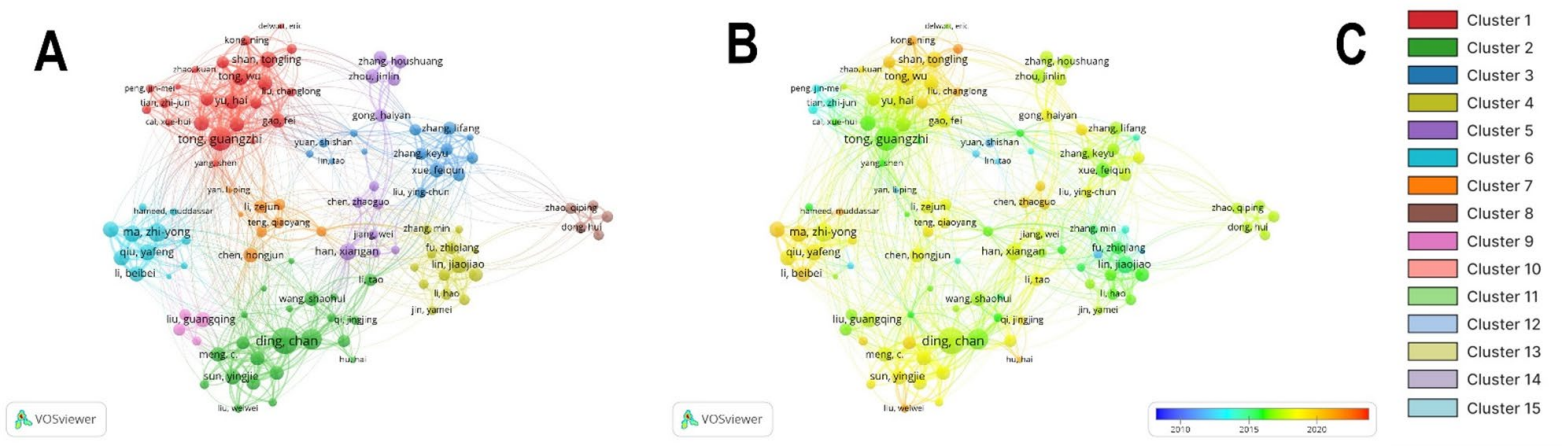
Co-authorship network analysis of SHVRI authors (2009–2023). The network generated using VOSviewer (v. 1.6.20, Leiden University), includes authors with a minimum of 10 documents and 300 citations. **A** Network visualization showing collaborative clusters; **B** Overlay visualization; **C** Color scale legend

The co-authorship analysis of the authors of SHVRI is presented in Fig. 3-A and B. Figure 3-C represents the color code for each cluster, respectively. These color codes are the same for all network visualizations in bibliographic coupling as well. It is conducted based on 10 documents with 300 citations per author, revealing 112 authors with 9 clusters with 1180 links and a total link strength (TLS) of 13,850. TLS shows the strength of collaborations among the authors. The most influential author in cluster 1 is Guangzhi Tong (207) who has strong ties with all the authors in cluster 1 except Eric Delwart (11). In cluster 2, Chan Ding (250) is the most influential author having strong ties with all the authors in his cluster. In cluster 3, Feiqun Xue (73) is the most influential author having strong ties with authors in this social network excluding Xing-Quan Zhu (13), and other authors who have formed another close social network inside the cluster. These authors include Shishan Yuan, (32), Zuzhang Wei (21), and Tao Lin (14). Cluster 4 shows Jiajiao Lin (104) as the most influential author having strong ties with all the authors in this cluster. In cluster 5, Xiangan Han (105) is the most influential. However, this cluster also has another social network inside it. Han has collaborative ties with all the authors except the social network, where the authors include Jinlin Zhou (80), Houshuang Zhang (65), Yongzhi Zhou (58), and Jie Cao (56). In cluster 6, Zhi-Yong Ma (122) is the most influential author, with strong ties to all authors in this cluster. In cluster 7, Hongjun Chen (75) is the most influential author; he also has strong ties with all the authors in his cluster. Cluster 8 is led by Qiping Zhao (48), having strong ties with all the authors in his cluster. In cluster 9, Guangqing Liu (81) is the most influential author due to the strength of ties with all the authors in this cluster. Since each cluster dictates a distinct social network. The top 15 authors, as per Table 3, are scattered in these clusters with Guangzhi Tong (207), Tongling Shan (92), Wu Tong (98), Yanjun Zhou (88), and Hai Yu (84) forming strong ties in cluster (1) Chan Ding (250) and Shengqing Yu (151) also form strong ties in cluster (2) Jiaojiao Lin (104) is present in cluster 4 while Xiangan Han (105) is in cluster 5. Cluster 6 has a dense network of Zhi-Yong Ma (122), Jian-Chao Wei (95), Yafeng Qiu (95), Ke Liu (83), Donghua Shao (82), and Beibei Li (82). Cluster 6, followed by cluster 5, has the most top authors, indicating strong collaboration. The largest contributions among the authors are from 2017 to 2020. Shishan Yuan (32) of cluster 3 is the earliest contributor (average publication year 2011). In contrast, Wen Zhang (36) is the most recent contributor (average publication year 2021).

**Fig. 4:**
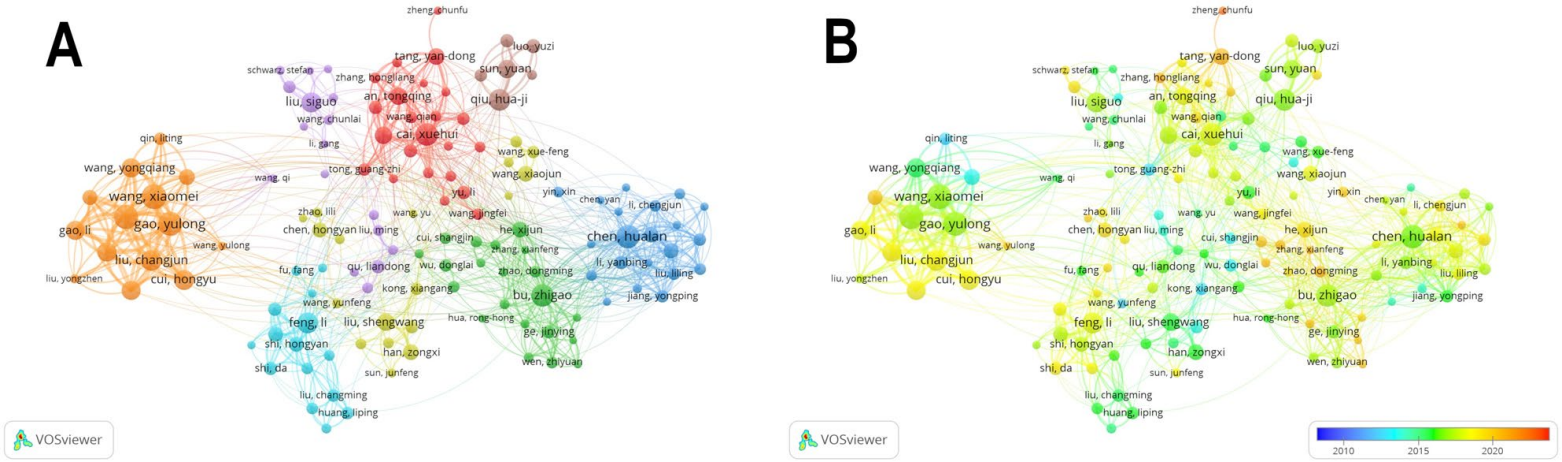
Co-authorship network analysis of HVRI authors (2009–2023). The visualization, generated via VOSviewer (v. 1.6.20, Leiden University), includes authors with a minimum threshold of 20 documents and 400 citations. **A** Network visualization of collaborative clusters; **B** Overlay visualization showing temporal distribution

The co-authorship analysis of HVRI is presented in Fig. 4-A and B, it is conducted on a minimum of 20 documents with 400 citations per author. It revealed 135 documents with 8 clusters with 1545 links and TLS 15,468. In cluster 1, Xuehui Cai (158) is the prominent node having connections with all the authors in the cluster except Chunfu Zheng (21). Zhigao Bu (158) is distinguished in cluster 2 due to his social network with all the authors in the cluster. Similarly, Hualan Chen (179) is also prominent in cluster 3. Cluster 4 is led by Shengwang Liu (96). However, this cluster has 2 social networks, the other led by Xiaojun Wang (78). Siguo Liu (116) dominates cluster 5 with another social network inside the cluster led by Liandong Qu (57). In cluster 6, Li Feng (134) is the prominent node having connections with all the authors in this cluster. Most of the top authors (9) from Table 3 are present in cluster 7, having a strong social network among themselves. Namely, these are Xiaomei Wang (184), Yulong Gao (178), Hongyu Cui (120), Xiaole Qi (171), Changjun Liu (129), Yanping Zhang (112), Yongqiang Wang (111), Li Gao (110), and Kai Li (109). This cluster is led by Xiaomei Wang. Cluster 8 is dominated by Hua-Ji Qiu (140), having strong connections within this cluster. The other top authors are scattered individually across other clusters. These include Xuehui Cai, in cluster 1, Hualan Chen, in cluster 3, Zhigao Bu, in cluster 2, and Li Feng, in cluster 6. The greatest number of contributions and collaborations are from the years 2012–2017. The earliest contributor is Liting Qin (46) (average publication year 2012) of cluster 7. In contrast, the most recent contributor is Chunfu Zheng, (21) of cluster 1 (average publication year 2021).

**Fig. 5:**
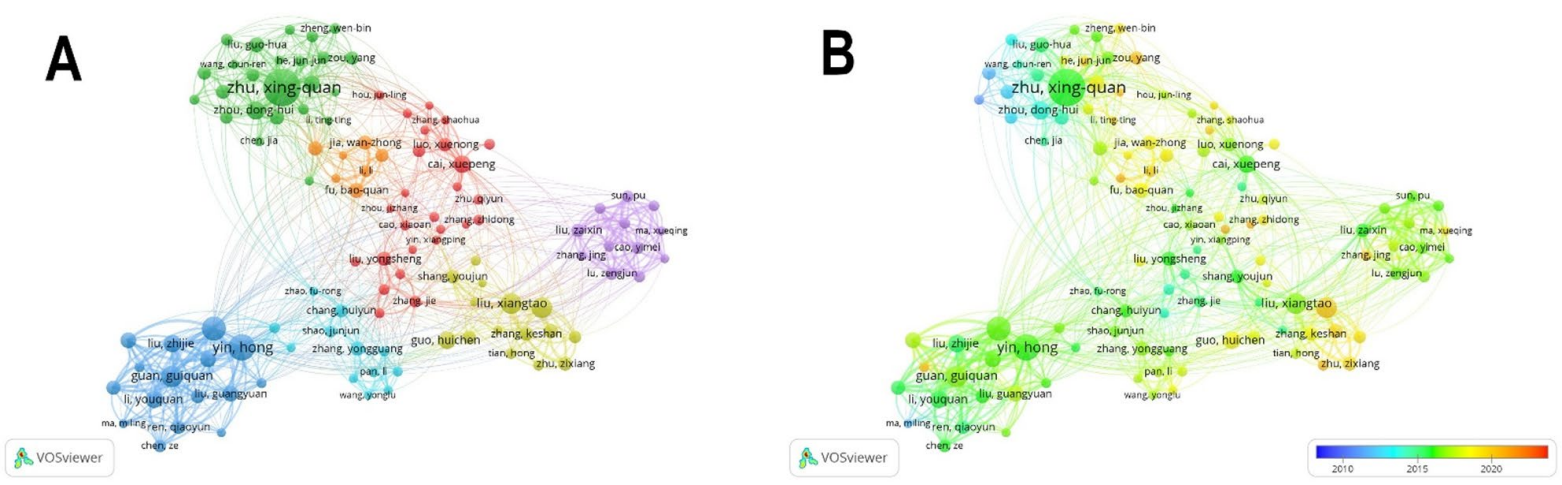
Co-authorship network analysis of LVRI authors (2009–2023). Data were visualized using VOSviewer (v. 1.6.20, Leiden University) based on a minimum threshold of 30 documents and 300 citations per author. **A** Network visualization showing collaborative clusters; **B** Overlay visualization illustrating temporal trends

The co-authorship analysis of LVRI is presented in Fig. 5-A and B, it is conducted based on 30 documents with 300 citations per author. It provided 112 authors with 7 clusters, links of 1299, and TLS 16,649. Cluster 1 is dominated by Xuepeng Cai (127), with links extending to all authors in the cluster except Yanmin Li (30), Guanglian Liu (33), Xiangping Yin (37), and Yuefeng Chu (40). Cluster 2 has the leading author, Xing-Quan Zhu (549), with links extending to all the authors in the cluster. Cluster 3 has the highest number of leading authors (8), as shown in Table 3. Namely, Hong Yin (279), Jianxun Luo (240), Guiquan Guan (185), Zhijie Liu (126), Youquan Li (124), Junlong Liu (106), Jifei Yang (105), and Guangyuan Liu (100). Hong Yin (279) is the leading author in cluster 3. Xiangtao Liu (178) is the leading author in cluster 4, having connections with all the authors in this cluster. Zaixin Liu (75) is the prominent node among the plethora of authors in cluster 5, having strong connections with all the authors in this cluster. In cluster 6, Yongguang Zhang (82) takes the lead, having almost all the connections in this cluster except Junzheng Du (38). Bao-Quan Fu (91) is the prominent node in cluster 7, with strong connections within the cluster. Other than cluster 3, the rest of the top authors, as per Table 3, are scattered as Xing-Quan Zhu, Dong-Hui Zhou (136), Hany M. Elsheikha (106) in cluster 2, Xiangtao Liu, Haixue Zheng (177), Huichen Guo (126) in cluster 4, and Xuepeng Cai, in cluster 1. Most of the collaboration was done between 2013 and 2019. The earliest contributor is Zi-guo Yuan (45) (average publication year 2011) of cluster 2, while the most recent contributor is Guanglian Liu (average publication year 2020) of cluster 1.

**Fig. 6:**
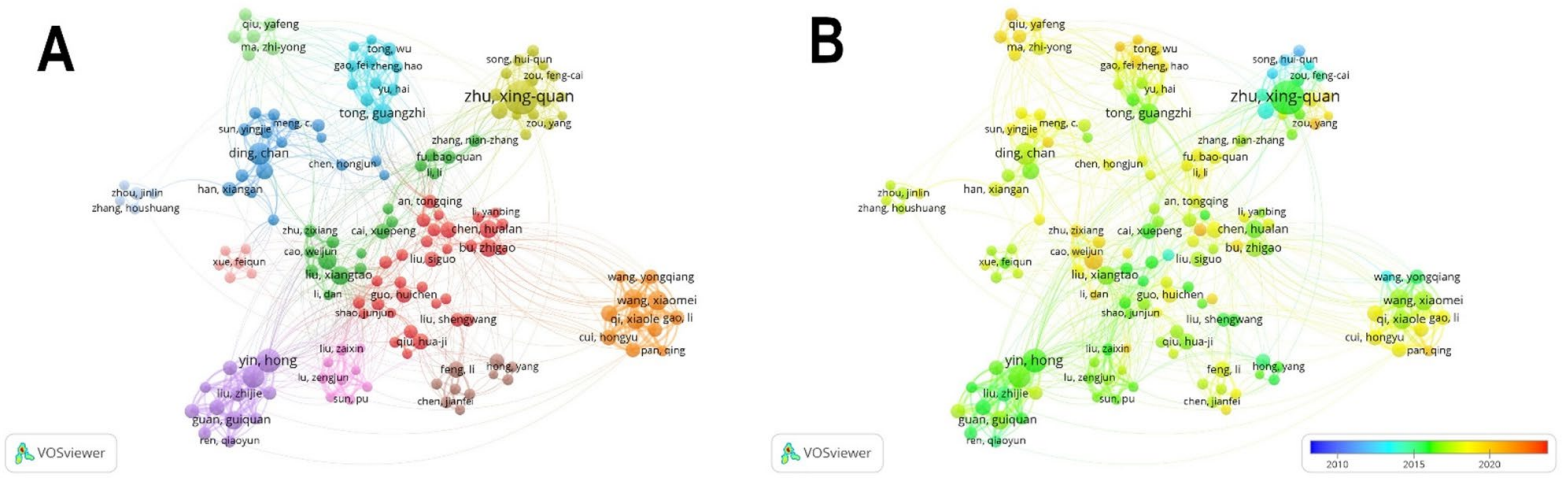
Co-authorship network analysis of CAAS-affiliated authors (2009–2023). The network was generated via VOSviewer (v. 1.6.20, Leiden University) using a minimum threshold of 50 documents and 500 citations. **A** Network visualization of collaborative clusters; **B** Overlay visualization showing temporal distribution of authorship

The combined co-authorship analysis of all the VRIs of CAAS is present in Fig. 6-A and B. The analysis was conducted based on at least 50 documents with 500 citations per author consequently giving 153 authors with links of 1298 and TLS 24,957. The analysis revealed distinct social networks among the authors, showcasing the collaborations across the VRIs.

In cluster 1, Hualan Chen (179), Zhigao Bu (158), Hua-Ji Qiu (140), Xuehui Cai (158), and Huichen Guo (126) are the most prominent nodes, respectively. In cluster 2, Xiangtao Liu (178), Haixue Zheng (177), and Xuepeng Cai (127) are the most prominent authors. In cluster 3, Chan Ding (250), Shengqing Yu (151), and Xiangan Han (105) are the most prominent nodes, while in cluster 4, Xing-Quan Zhu (549) and Dong-Hui Zhou (136) are the most distinguished nodes. Hong Yin (279) is the most prominent in cluster 5, followed by Jianxun Luo (240) and Guiquan Guan (185). The internal strength of this social network is truly remarkable. In cluster 6, Guangzhi Tong (207) is distinguished while in cluster 7, Xiaomei Wang (184), Yulong Gao (178), and Xiaole Qi (171) are the most dominant nodes. Their internal strength rivals that of cluster 5 in being the most extraordinary. Cluster 8 is led by Li Feng (134) and Jiaojiao Lin (104). Both of these authors lead distinct social networks in this cluster. Zaixin Liu (75) led cluster 9 while Feiqun Xue (73) led cluster 10. In cluster 11, Zhi-Yong Ma (122) is prominent, while in cluster 12, Jinlin Zhou (80) is the central author. In general, Xing-Quan Zhu, of cluster 4, Hong Yin, of cluster 5, Chan Ding, of cluster 3, and Guangzhi Tong, of cluster 6, are the most dominant nodes among all others. Their dominance is dictated by the strength of their connections among the clusters. Most contributions and collaborations are from the years 2015–2018. The earliest contributor is Rui-qing Lin (77) (average publication year 2011) of cluster 4, while the most recent contributor is Yan-dong Tan (86) (average publication year 2020).

### Thematic map

A strategic map illustrates a theme’s internal strength and importance based on the theme’s density (vertical axis) and centrality (horizontal axis). It involves the clustering of the trigrams extracted from the abstracts, which are then mapped into quadrants [53]. These are divided into four quadrants, each showing the degree of development and importance of the theme in the broader field. Each of the four quadrants represents a state of the theme. For instance, quadrant 1 shows the motor themes, which show the advanced state, indicating high research input and relevant importance to the field. These can also be known as the core themes. Quadrant 2 shows the niche themes, which are highly researched and specialized to specific disciplines, geographic areas, or industries rather than being broadly applicable. Quadrant 3 shows the emerging or declining themes, which either decline or move into a different quadrant over time. These are also known as transitioning themes. Quadrant 4 shows the basic themes, which contain the foundational knowledge in the broader field [54]. The current analysis of each VRI is based on abstracts to answer RQ7.

The strategic analyses of SHVRI, HVRI, and LVRI were mapped based on abstracts. Trigrams, along with word stemming (the parameter set to yes), were used. A synonym-reducing file was used to reduce the redundancy of the terms. The label size of 0.05 and 30 words was used.

**Fig. 7:**
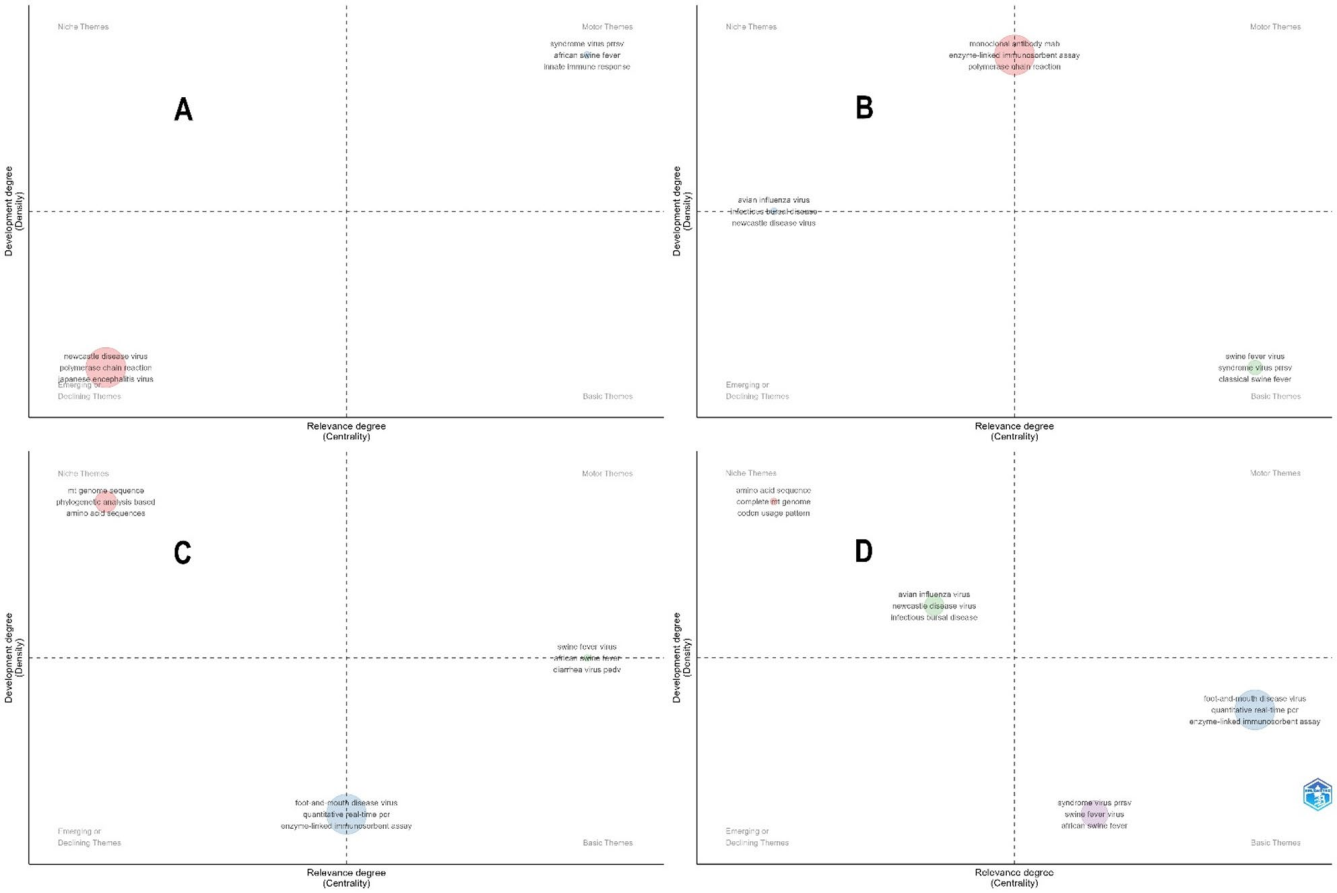
Thematic mapping of research trends based on article abstracts (2009–2023). Maps were generated using the Biblioshiny package in R, utilizing trigram analysis, word stemming, and a synonym-reduction filter. Parameters include a minimum word frequency of 30 and a label size of 0.05. The quadrants are defined by centrality (horizontal axis) and density (vertical axis) for: **A** SHVRI, **B** HVRI, **C** LVRI, and **D** CAAS

The motor and emerging or declining themes are represented here as seen in Fig. 7-A, which may show the core and highly developed research agenda. The motor themes include “syndrome virus prrsv”, “african swine fever”, and “innate immune response”. The presence of “newcastle disease virus”, “polymerase chain reaction”, and “japanese encephalitis virus” represents that these themes may be waning in interest or are at an early developmental stage. The direction of these themes indicates SHVRI’s interest in disease-specific research rather than a dispersed foundational focus.

The HVRI map is depicted in Fig. 7-B. It shows the presence of “swine fever virus”, “syndrome virus prrsv”, and “classical swine fever” in the basic themes. The viruses, such as “avian influenza virus”, “infectious bursal disease”, and “newcastle disease virus”, are attributed to emerging or declining themes. Their positioning indicates that they may be declining or emerging, but are underdeveloped. The “monoclonal antibody mab”, enzyme-linked immunosorbent assay”, and “polymerase chain reaction” are present in the motor themes, which indicate HVRI’s technical strength in specialized diagnostic tools.

In the case of LVRI, Fig. 7-C shows that “foot-and-mouth disease virus”, “enzyme-linked immunosorbent assay”, and “quantitative real-time pcr” are present in emerging or declining themes. Their positioning suggests a declining phase, possibly reflecting nascent interest. The basic themes consist of “swine fever virus”, “african swine fever”, and “diarrhea virus pedv”. The trigrams of “mt genome sequence”, “phylogenetic analysis based”, and “amino acid sequences” belong to the niche themes. LVRI focused mostly on genetic analyses linked to pathogens.

The overall analysis of the VRIs of the CAAS is illustrated in Fig. 7-D. It reveals that “foot-and-mouth disease virus”, “quantitative real-time pcr”, and “enzyme-linked immunosorbent assay” are present in basic themes, along with “syndrome virus prrsv”, “swine fever virus”, and “african swine fever”, indicating work on the foundational bases that play a major role in the broader fields. The niche themes contain “complete mt genome”, “amino acid sequence”, and “codon usage pattern”, along with “avian influenza virus”, “newcastle disease virus”, and “infectious bursal disease”, representing specialized research focus. Most of the work attributed to niche and basic themes indicates that specializations regarding foundational areas were generally focused.

### Bibliographic coupling

The bibliographic coupling of documents shows that the articles with shared literature references share a common theme as well S Kumar, N Pandey and A Haldar [55]. Based on this premise, articles are grouped into distinct clusters. These clusters contain documents that vary in size depending on the number of articles they contain [53]. The clusters contain articles that show the core theme. These themes are representatives of the research focuses of VRIs of the CAAS. The following coupling analyses reveal each institute’s core themes and a generalized observation to address *RQ8.*

#### SHVRI

**Fig. 8:**
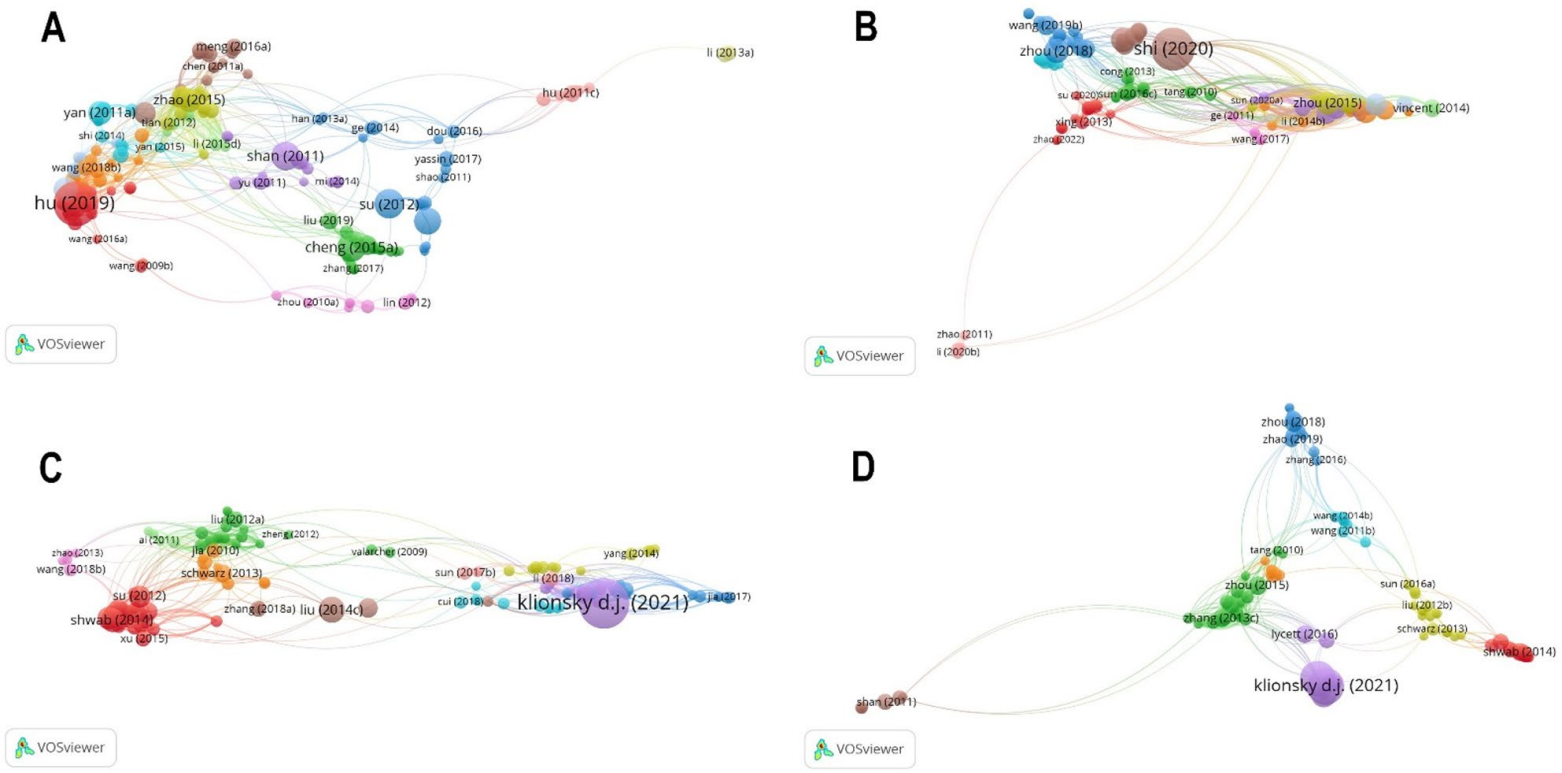
Bibliographic coupling of authors (2009–2023) across the four studied institutions. The networks, generated via VOSviewer (v. 1.6.20, Leiden University), illustrate the similarity between authors based on shared cited references. Panels show network visualizations for: **A** SHVRI, **B** HVRI, **C** LVRI, and (**D**) CAAS

The coupling analysis of documents revealed 191 articles, leading to 13 clusters based on a criterion of 30 citations per document. The total number of links received is 895, and TLS 2217 as illustrated in Fig. 8-A. A paper was removed from the coupling analysis of SHVRI and CAAS of DJ Klionsky, K Abdelmohsen, A Abe, MJ Abedin, H Abeliovich, A Acevedo Arozena, H Adachi, CM Adams, PD Adams, K Adeli, et al. [56], due to its overwhelming presence, clouding the other nodes in the analysis.

##### Cluster 1

Newcastle disease virus is the core theme. Most papers discuss apoptosis and autophagy in Newcastle disease virus. The impacts of Newcastle disease on the immune system, along with vaccine interventions such as DNA-encapsulated, quaternized chitosan nanoparticles, etc. The highly cited article (665) in this cluster discusses the role of CHOP in apoptosis [43].

##### Cluster 2

*Schistosoma japonicum* is the core theme in cluster 2. It’s proteomics analysis, surveillance, diagnosis, effect on the immune system, host-immune responses, and parasite-host interactions. The highly cited article (278) in this cluster examines the role of miRNA in cancer diagnosis [57].

##### Cluster 3

*Toxoplasma gondii* is the core theme, focusing on genotyping, serological detection, isolates, prevalence, and zoonosis. The second sub-theme in this cluster concerns Escherichia coli, including its genetic characterization, antimicrobial resistance, foodborne zoonotic disease, and virulence. The highly cited article (291) in this cluster discusses the ancestral lineages of *Toxoplasma gondii* [44].

##### Cluster 4

Porcine reproductive and respiratory syndrome virus (PRRSV) is the core theme of cluster 4. This cluster contains the articles related to origin, detection, genetic characterization, geographical distribution across China, phylogenetic analysis, vaccine interventions like attenuated live vaccine, and the newly emerged NADC-30-like strain of the PRRSV. The highly cited article (184) in this cluster discusses the factors responsible for the emergence of highly pathogenic PRRSV in China [58].

##### Cluster 5

The influenza virus is the core theme. However, most of the discussion revolves around the 2009 pandemic H1N1 virus, H5N5, and H3N2 viruses, comprising genes of the 2009 pandemic H1N1 virus, H9N2, and zoonotic threats of H9N2 from pigs to humans. Moreover, subthemes discussed here include cryptosporidium, bovine viral diarrhea, porcine parvovirus (PPV4), porcine bocavirus, and porcine circovirus. The highly cited article (273) in this cluster examines the virome present in the feces of pigs in a high-density farm [59].

##### Cluster 6

Duck tembusu virus is the core theme. It’s vaccine development, transmission, prevalence, and detection. Japanese encephalitis virus is the subtheme. The highly cited article (193) in this cluster investigates the emergence of a new strain of duck tembusu virus in mainland China [60].

##### Cluster 7

The core theme is the innate immune system. However, to be more specific, the relation is to the studies of innate immune response to viral infections in chickens. Many articles discuss the STING (stimulator of interferon genes) and MAVS (mitochondrial antiviral signaling) in correspondence with antiviral immune responses, the mechanism involved with IFN (interferon) gene activation, and molecular signaling pathways (IRF 7). The highly cited article (93) in this cluster examines the inhibition of the STING pathway by ASFV [61].

##### Cluster 8

Pseudorabies virus is the core theme. Its viral pathogenesis, host immune responses, vaccine development, like bartha-k61, and molecular characterization. The highly cited article (169) in this cluster studies the genome-wide CRISPR screen and identifies the factors through which the host becomes susceptible to sars-cov-2 [62].

##### Cluster 9

*Eimeria tenella* is the core theme. Most of the papers discuss the identification, molecular characterization, and diclurazil treatments. The highly cited article (69) in this cluster represents a comparative analysis specifically targeting 529-bp repeat element for toxoplasmosis diagnosis [63].

##### Cluster 10

*Riemerella anatipestifer* is the core theme. its detection, identification, genetic characterization, vaccine development (inactivated), and pathogenicity. The highly cited article (98) in this cluster discusses OMPA as a potential factor for the virulency of *Riemerella anatipestifer* [64].

##### Cluster 11

The core theme in this cluster is PRRSV. However, since cluster 4 already discusses the same theme. The subtheme of duck parvovirus is unique here. The research is mostly on its pathogenesis, vaccine development, molecular characterization, and epidemiology. The highly cited article (60) in this cluster discusses the role of host miR-26a in PRRSV infection [65].

##### Cluster 12

The porcine epidemic diarrhea virus is the core theme here. Its molecular characterization and evolution, pathogenesis and viral mechanisms, host-immune response, vaccine development and its efficacy. The highly cited article (198) in this cluster discusses the factor that suppresses liver cancer cell growth [66].

##### Cluster 13

*Staphylococcus aureus* is the core theme. The aspects studied in this cluster include the spectinomycin resistance gene identification, detection of a resistant gene in methicillin-resistant *Staphylococcus aureus*, and identification of multidrug-resistant genes in staphylococci. The highly cited article (81) in this cluster identifies the Isa (E) gene in MRSA (methicillin-resistant *Staphylococcus aureus*) strain isolated from swine [67].

#### HVRI

The coupling analysis of HVRI was performed using at least 60 citations per document, revealing 126 articles. The connected articles were used for this analysis. A total of 12 clusters, 890 links, and TLS 2707 were obtained as shown in Fig. 8-B.

##### Cluster 1

The core theme of the porcine epidemic diarrhea virus is that the majority of articles conduct studies on this virus. Studies on this virus mainly focus on its epidemiology and genetic diversity in China. The highly cited article (141) in this cluster discusses the model where the function of the open reading frame 3 (orf3) gene of the porcine epidemic diarrhea virus [68].

##### Cluster 2

The core theme of cluster 2 is pseudorabies. The genetic characterization and epidemiology of pseudorabies are discussed here. The subthemes, along with pseudorabies, are also persistent in this cluster, which include infectious bronchitis virus and avian influenza virus. The highly cited article (154) of this cluster discusses the challenges and opportunities that are faced with the control of swine pseudorabies in China [69].

##### Cluster 3

This cluster mainly focuses on the African swine fever virus as its core theme. The focus on the prevalence and emergence in China along with genetic characterization is inferred in this cluster. The highly cited paper (423) of this cluster evaluates the emergence of the African swine fever virus in China during 2018 [70].

##### Cluster 4

This cluster has the core theme of the H7N9 influenza virus. This cluster revolves around the strains of the influenza virus, mostly H7N9, its zoonosis, pathogenicity, and effective control strategies. The subtheme of this cluster includes H5N6 strain of influenza virus. The highly cited article (364) in this cluster demonstrates how MIR2911 (microRNA) efficiently suppresses the viral infection [71].

##### Cluster 5

This cluster majorly discusses the core theme of the H5N1 strain of influenza virus. Its transmission, virulence, characterization, and evolution are revolving in this cluster. The highly cited article (334) in this cluster identifies the amino acids responsible for transmission of H5N1 to mammalian hosts [72].

##### Cluster 6

This cluster has the core theme of PRRSV. The geographical prevalence, emergence, along immune responses pertain to this cluster. The highly cited article (184) discusses the factors responsible for the recent emergence of PRRSV in China [58].

##### Cluster 7

This cluster discusses the vaccine for the avian influenza virus as a core theme. The subthemes in this cluster are related to the duck genomic characterization concerning influenza virus, and the emergence of avian influenza virus, mostly in wild birds. The highly cited article (335) in this cluster discusses the role of migratory birds like ducks, swans, and geese in the spread of the influenza virus recently [73].

##### Cluster 8

Cluster 8 discusses the sars-cov 2 as its core theme. The factors that revolve around its inhibition, antibody responses, susceptibility, transmission, and epidemiology are discussed in this cluster. The most cited article (1317) in this cluster shows the susceptibility of domesticated animals along with dogs, cats, and ferrets to sars-cov 2 [74].

##### Cluster 9

This cluster mainly focuses on the efficacy of recombinant vector vaccines. These include the goat poxvirus-vectored peste des petits ruminants vaccine, Newcastle disease virus-vectored rabies and nipah encephalitis vaccines, recombinant VSV-vectored MERS-CoV vaccine, and novel chimeric virus-like particles vaccine. The highly cited article (86) in this cluster discusses the efficacy of Newcastle disease virus-vectored rabies vaccine for dogs and cats [75].

##### Cluster 10

This cluster has the core theme of chitosan nanoparticles. The discussion here focuses on how the preparation of the Newcastle DNA vaccine encapsulated in chitosan nanoparticles enhances its efficacy. The highly cited article (116) in this cluster is a review that explains the importance of mucosal vaccines and their limited availability as a potential challenge [76].

##### Cluster 11

This cluster pertains to the swine influenza virus as the core theme. Its virulence, transmissibility, prevalence, and surveillance of the swine influenza virus. The surveillance, primarily focused worldwide, especially in European countries, is emphasized in this cluster. The highly cited article (224) of this cluster highlights the need for surveillance and research focus on the swine influenza virus worldwide [77].

##### Cluster 12

This cluster has only 2 articles where the theme represented is respiratory droplets. The transmission of H7N9 to ferrets [78] by the highly cited article in this cluster (356) and H5N1 (hybrid having the H1N1 genes) to guinea pigs [79] by respiratory droplets is discussed here.

#### LVRI

The coupling analysis of LVRI was performed using the criteria of at least 50 citations per document resulting in 124 articles providing 11 clusters. The total links are 649, and TLS is 1992 as depicted in Fig. 8-C.

##### Cluster 1

This cluster has the core theme of *Toxoplasma gondii*, with emphasis on vaccines, epidemiology, genetic characterizations, zoonotic effects, and trials conducted on mice in producing protective immunity. The highly cited article (327) in this cluster examines the geographical patterns of *Toxoplasma gondii* [80].

##### Cluster 2

It shows the core theme of the mitochondrial genome, with majority of the articles working on genetic characterization and comparative analysis. The genetic characterization studied through mitochondrial genome in these articles is of live flukes *Opisthorchis viverrini* and *Clonorchis sinensis*, ticks from Southwestern Romania, six *Eimeria* species namely *E. acervulina*,* E. brunetti*,* E. maxima*,* E. necatrix*,* E. tenella* and *E. praecox*, 3 cestode species of *Taenia* tapeworms which include *Taenia multiceps*,* T. hydatigena*, and *T. taeniaeformis*, 2 whipworms which include *Trichuris ovis* and *Trichuris discolor*, 3 parasitic nematodes of birds which include *Ascaridomorpha*,* Rhabditomorpha*, and *Diplogasteromorpha*, and brown dog tick namely *Rhipicephalus sanguineus* while the comparative analysis is of *Ascaris lumbricoides* and *Ascaris suum* from pigs and humans, and *Trichuris* of humans and pigs. All these analyses are performed on mitochondrial genomic-based datasets. The highly cited article (159) in this cluster discusses the identification and co-infection rates of *Anaplasma ovis*,* A. bovis*, and *A. phagocytophilum* [81].

##### Cluster 3

This cluster has mainly pertained to African swine fever, the role of structural proteins in virulence, the interaction of the virus with the host immune system, and efforts towards vaccine development. The highly cited article (140) in this cluster discusses the function that USP13 plays in innate antiviral immunity [82].

##### Cluster 4

This cluster has the core theme of microRNA. Their role in autoimmune diseases, cancer, host genes, and parasites. Codon usage pattern as a subtheme is indicated where some of the studies include dengue virus, hepatitis C virus, enterovirus, *Taenia saginata*, etc. The highly cited article (98) discusses the role of microRNAs in parasites and the infections associated with them [83].

##### Cluster 5

This cluster has the foot and mouth disease virus as the core theme. Most of the work in this cluster revolves around the interaction of the viruses with cellular processes, including apoptosis and immune response modulation. The highly cited article (1494) in this cluster is the critical assessment of autophagy [84].

##### Cluster 6

This cluster shows vaccines as a core theme with articles studying foot and mouth disease vaccines, virus-like particles as vaccines, mucosal vaccines, DNA vaccines, and epitope-based vaccines. The highly cited article (98) in this cluster discusses the role of flagellin and its use as a potential vaccine [85].

##### Cluster 7

This cluster has parasitic diseases as the core theme, namely, some of these are toxocariasis, *Taenia multiceps*, sparganosis, etc. The articles in this cluster predominantly explore the host-parasite dynamics, epidemiology, and genomics of parasitic diseases. The highly cited article (186) in this cluster examines the genomic characteristics of *Haemonchus contortus* [86].

##### Cluster 8

This cluster focuses on zoonotic diseases as the core theme. Some of these include avian influenza, thrombocytopenia syndrome, etc. The highly cited article (405) in this cluster discusses the thrombocytopenia syndrome and its potential as a tick-borne zoonotic disease [87].

##### Cluster 9

This cluster shows cryptosporidium as the core theme. Its prevalence, genetic characterization, and zoonosis. The highly cited article (112) elaborates on the prevalence of cryptosporidium in HIV-infected people [88].

##### Cluster 10

This cluster represents the Seneca Valley virus, its emergence, genetic characteristics, and surveillance. The highly cited article (120) summarizes the recent progress in type I interferons [89].

##### Cluster 11

This cluster focuses on the Fasciola species as the core theme. The essays for their identification and differentiation. Moreover, their genetic characterization, mitochondrial genomes, and comparative analysis. The highly cited article (81) discusses the assessment of *Fasciola* and *Fasciola gigantica* along with their comparative analysis with *F. hepatica* [90].

#### CAAS

The bibliographic coupling analysis of all the VRIs of the CAAS revealed the key themes which were researched by all the institutes (Fig. 8-D). At a criterion of 90 citations, analysis included 105 documents, which collectively exhibited 419 link strength, and 1524 TLS.

##### Cluster 1

The core theme of cluster 1 is *Toxoplasma gondii*. Most articles discuss the diagnosis, epidemiology, genetic characterization, global diversity, prevalence, transmission, vaccines, and zoonosis. The highly cited article (327) in this cluster examines the genetically diverse geographical patterns of *Toxoplasma gondii* [80].

##### Cluster 2

The Avian influenza virus is the core theme of cluster 2. More specifically, the emphasis is on the H5N1 strain of the influenza virus, its transmission, evolution, outbreaks, virulence, and control. The subtheme of cluster 2 is related to the H7N9 strain of the avian influenza virus. The highly cited article (364) in this cluster shows that MIR2911 targets the influenza A virus [71].

##### Cluster 3

African swine fever virus is the core theme. The studies concerning the core theme include genetically modified live attenuated vaccine, pathogenicity, regulation of sting-mediated signaling pathway, emergence, prevalence, challenges ASF presented to China, and architecture of ASF. The highly cited article (423) in this cluster investigates the emergence of ASF in China in 2018 [70].

##### Cluster 4

Cluster 4 represents the theme of molecular and genetic studies of animal pathogens. The reason for this theme is due to most articles discussing it directly or indirectly. There is no definitive mention of a repetitive theme within the titles. The aspects studied are molecular epidemiology, such as porcine epidemic diarrhea virus and anaplasma species, genetic characterization, such as of *Taenia* species and *Ascaris*, zoonotic pathogens, and other pathogens like senecavirus A, porcine epidemic diarrhea virus, and avian leukosis. The highly cited article (186) in this cluster discusses the genomic development of *Haemonchus contortus* [86].

##### Cluster 5

Represents SARS-CoV-2 as its core theme. Most of the studies correlate to the control measures against SARS-CoV-2, such as clofazimine, susceptible animals like ferrets, cats, dogs, and domestic animals, and antibodies associated with SARS-CoV-2, like human monoclonal antibodies, and vaccines like adenovirus vectored vaccine. The highly cited article (1494) in this cluster presents work on autophagy [84].

##### Cluster 6

It shows the pseudorabies virus as the core theme. Aspects of the core theme studied in this cluster include its pathogenicity, genetic characterization, control, emergence, and immune responses. The Subtheme is the foot-and-mouth disease virus. The highly cited article (164) in this cluster discusses the effects on the immune system by the foot-and-mouth disease virus [91].

##### Cluster 7

It represents microRNAs as the core theme. Particularly, their role in cancer, radiation-induced bystander effect, and parasites, along with parasitic infections. The highly cited article (278) in this cluster discusses the role of miRNAs in cancer [57].

##### Cluster 8

It shows porcine circovirus, more specifically types 2 and 3, as its core theme. The genetic characteristics and occurrence are mostly represented here in correlation with the theme. The highly cited article (273) in this cluster examines the virome in the feces of pigs in high-density farm [59].

## Discussion

This bibliometric analysis investigates the productivity and trends of the VRIs of the CAAS. The analysis reveals that each VRI shows a general upward trend in the NP over the years. However, HVRI published the most articles each year, followed by LVRI, and then SHVRI. In terms of TC, HVRI generally received the most, followed by LVRI and then SHVRI. In terms of the h-index, SHVRI peaked in 2015, while HVRI peaked in 2013, and LVRI in 2016. While LVRI leads in total publications volume, HVRI exhibits a “quality over quantity” trajectory, which is represented by the highest total citation and h-index. The reason may be attributed to two factors, one is the presence of Hualan Chen, who achieved an h-index of 52 despite having significantly fewer publications than the other leading authors, and secondly, HVRI’s focus on emerging viral threats led to hallmark publications in high impact journals such as Science, notably the 2020 study on SARS-CoV-2 susceptibility in domestic animals which garnered over 1300 citations. In general, the h index seems to have followed a stable path with slight fluctuations, which is not unusual, as the h index is not simply a measure of citations; rather, it reflects the spread of citations across publications. The top-cited articles for each VRI indicate the key focus of the top papers. SHVRI strongly emphasizes virology and parasitology research, as evidenced by publications on Newcastle disease virus and *Toxoplasma gondii*. The top-cited article in SHVRI is a comprehensive review of autophagy [56]. HVRI mostly focused on emerging viral threats, including COVID-19 research and avian influenza. The top-cited article in HVRI discusses the susceptibility of domesticated animals and ferrets, dogs, and cats to SARS-CoV-2 [74]. LVRI focuses mostly on parasitology and genomics. The highly cited article also discusses the comprehensive review of autophagy in a new edition [84]. The most interesting point to note in the citation architecture is that methodological guidelines often overshadow disease-specific research in academic influence. For instance, the most frequently referenced article for both SHVRI and LVRI was a review focused on autophagy monitoring assays that received over 4,000 citations. The reason such influential technical treatises attract so many citations is that they serve as cross-disciplinary standards that cannot be ignored. Meanwhile, research focusing on specific pathogens, like *Schistosoma japonicum* or foot-and-mouth disease virus, is usually limited to the specialized veterinary or parasitological communities. The institutional impact analysis shows that there are certain peaks in time, which are directly associated with the publication of high-impact papers as hallmarks. In the case of SHVRI, the peak of exceptionally high citations in 2016 (6,687 citations) is largely contributed to by a single landmark article, the 3rd edition of the “Guidelines for the use and interpretation of assays for monitoring autophagy”. This review alone has accumulated 4347 citations, demonstrating that total citation metrics for a given year can be skewed by a foundational technical standard. Contrarily, the fact that SHVRI reached its highest h-index in 2015 (h-index 27) indicates that the research reported in 2015 had a more balanced and pervasive impact on a wider set of articles, rather than being concentrated in a single outlier. Similarly, HVRI had its most active year in 2013 (h-index 37), with an overall citation growth of its fundamental viral research, and in 2020, it experienced a citation burst as a result of urgent COVID-19 research, including the Science article on SARS-CoV-2 susceptibility in domestic animals, which had 1,317 citations. Veterinary Microbiology is the leading publication venue for SHVRI and HVRI, indicating its importance as a publication outlet. However, in the case of LVRI, Parasites and Vectors is the leading journal. The Journal of Virology has demonstrated significant influence, with a high h-index for publications on SHVRI and HVRI, while maintaining a decent h-index in LVRI. The most influential authors include Chan Ding, from SHVRI, Xiaomei Wang, from HVRI, and Xing-Quan Zhu, from LVRI. Their contributions are currently at the top of their respective institutes. The keyword analysis showed the most frequently occurring keywords. The dominant keywords for SHVRI are “*Schistosoma japonicum*”, “virus”, “newcastle disease virus”, “prrsv”, “pathogenicity”, and “*Eimeria tenella*”. HVRI’s word cloud emphasizes “pathogenicity”, “vaccine”, “porcine reproductive and respiratory syndrome virus”, “monoclonal antibody”, “classical swine fever virus”, and “african swine fever virus”. The most prominent keyword for LVRI is “*Toxoplasma gondii*”, followed by “fmdv”, “china”, “prevalence”, “phylogenetic analysis”, and “vaccination”. In general, CAAS shows emphasis on “*Toxoplasma gondii*”, “fmdv”, “phylogenetic analysis”, “vaccination”, “prevalence”, “pathogenicity”, and “immune responses”. These clouds suggest that parasitology, virology, and epidemiology are the major research themes across the CAAS VRIs.

The trend analysis of titles identifies the research topics that have gained prominence over the years. In the case of SHVRI, from 2015 to 2020, “newcastle disease virus” gained dominance. However, “respiratory syndrome virus” was another topic of interest from 2012 to 2018. The most recent trend, from 2020 to 2023, reveals a growing emphasis on the “swine fever virus”. These indicate a shift in research interests. HVRI’s trend topics are heavily centered around viral diseases, with “swine fever virus” gaining prominence in research activity from 2015 to 2022, with “avian influenza virus” being another major research interest from 2013 to 2021. “newcastle disease virus” also received significant attention from 2014 to 2018. “foot-and-mouth disease virus” emerged as a dominant trend for LVRI from 2014 to 2020. In a similar vein to SHVRI, a notable increase in research on “swine fever virus” has been observed in LVRI since 2021 as well. “*Toxoplasma gondii* infection” was a key research focus from 2014 to 2018. In general, CAAS showed highly impactful work with a special focus on viral diseases, particularly those affecting swine. “swine fever virus” had substantial research output, especially from 2014 to 2022. “foot-and-mouth disease virus” was of major interest from 2014 to 2020 and research on “african swine fever” intensified from 2021 to 2023 indicating a response to its recent emergence. This strategic redirection of resources from Newcastle disease virus (2020–2023) to Swine fever virus (2020–2023) reflects a response to the national biosecurity emergencies. This transition was necessitated by the 2018 African Swine fever in PR China, which posed a severe threat to the food security and livestock sector, and the fact that such a trend is monitored by all 3 VRIs shows CAAS’s rapid response as a national research team. Furthermore, “respiratory syndrome virus” had a major trend from 2012 to 2020. These indicate the VRIs commitment to addressing animal health challenges and their contribution to the control of diseases.

The co-authorship analysis of SHVRI revealed 9 distinct clusters, with Guangzhi Tong and Chan Ding as the most prominent nodes. Cluster 6 contained some of the top authors with a strong collaborative network. The central figures around HVRI are Cai, Xuhui, Zhigao Bu, and Hualan Chen. Moreover, among the total 8 clusters, cluster 7 contains the largest number of collaborating top authors. In the case of LVRI, there are 7 clusters obtained with Xing-Quan Zhu as the most dominant node. The overall analysis of the VRIs of the CAAS reveals 12 clusters with major interconnectivity among the institutes. Xing-Quan Zhu, Hong Yin, Chan Ding, and Guangzhi Tong are the most influential among the entire network of authors. The strategic map was conducted based on abstracts, providing an important aspect of the research foundations. SHVRI has focused mostly on core themes and emerging or declining themes. The dominant motor themes include “syndrome virus prrsv”, “african swine fever”, and “innate immune response”, while the emerging or declining themes include “newcastle disease virus”, “polymerase chain reaction”, and “japanese encephalitis virus”. HVRI’s thematic maps emphasize viral diseases, which are consistent with the findings on popular keywords and trend topics. The focus of this institute is on viral diseases affecting swine and poultry. These are evident from the basic themes, which majorly include “swine fever virus”, “syndrome virus prrsv”, and “classical swine fever”, and emerging or declining themes, which include “avian influenza virus”, “infectious bursal disease”, and “newcastle disease virus”, which indicate research focus and potential shifts. The terms “monoclonal antibody mab”, “enzyme-linked immunosorbent assay”, and “polymerase chain reaction” appear in the motor themes, showcasing HVRI’s expertise in specialized diagnostic tools. LVRI has provided a diverse research portfolio focusing mostly on genetics, immunology, and virology. The most frequent terms that point towards genetics are “mt genome sequence”, “phylogenetic analysis based”, and “amino acid sequences” in niche themes, while the basic themes indicate a strong focus on swine fever viruses, more specifically, “swine fever virus”, “african swine fever”, and “diarrhea virus pedv” as the prominent topics. Moreover, the emerging or declining themes are “foot-and-mouth disease virus”, “quantitative real-time pcr”, and “enzyme-linked immunosorbent assay”. The research priorities of the VRIs align closely with the global “One Health” framework, with extensive research on zoonotic pathogens such as *Toxoplasma gondii*, Avian influenza, and SARS-CoV-2. Alternatively, there is an observable divergence as antimicrobial resistance (AMR) in zoonotic pathogens was not observed as an integral part of the research focus during the 2009–2023 period. Overall, CAAS reinforces the general key research topics, including virology, immunology, and molecular biology sections.

CAAS includes a diverse key research focus by assimilating major areas in each institute. The coupling analysis includes groupings that provide the main research focuses of each institute. SHVRI’s research interests include animal diseases, pathogens, viral diseases, and parasites. Key themes of SHVRI include Newcastle disease virus, *Schistosoma japonicum*, *Toxoplasma gondii*, porcine reproductive and respiratory syndrome virus (PRRSV), influenza virus, duck tembusu virus, innate immune responses to viral infections, pseudorabies virus, *Eimeria tenella*, *Riemerella anatipestifer*, porcine epidemic diarrhea virus, and *Staphylococcus aureus*. HVRI focuses mostly on viral diseases, particularly those of swine and poultry. The key themes of HVRI include porcine epidemic diarrhea virus, pseudorabies, African swine fever virus, various strains of influenza virus (H7N9, H5N1, and swine influenza), recombinant vector vaccines, and chitosan nanoparticles for vaccine delivery. LVRI explored diverse topics such as parasites, viruses, and vaccines. Their key themes include *Toxoplasma gondii*, mitochondrial genome analysis of various parasites, African swine fever, microRNA, foot-and-mouth disease virus, vaccines (including those for foot-and-mouth disease and mucosal vaccines), parasitic diseases, zoonotic diseases, Cryptosporidium, Seneca Valley virus, and Fasciola species. The thematic focus on specific pathogens in the VRIs is a direct reflection of the national priorities in agriculture, economic interests, and biosecurity needs. The high prevalence of *Schistosoma japonicum* in the academic production of SHVRI is predetermined by its endemic status in China, which requires persistent scientific activity to protect the population and preserve the agricultural system [92, 93]. The publicity surrounding *Toxoplasma gondii*, notably within the context of the research in the LVRI, is being driven by the zoonotic interest and the high prevalence rate in the Chinese population, thus heralding the need to develop effective vaccines and diagnostic tools [94, 95]. Furthermore, the significant increase in the research on African swine fever, as indicated by the trend-topic shifts between 2021 and 2023, is a strategic reaction to a national crisis. Since the entry of the virus into China in 2018 and its rapid spread, African swine fever has become a disastrous issue to the swine industry and food security nationwide [96, 97] thus warranting its being part of the funding and research priorities of CAAS. In general, CAAS highlights the major focus of each institute, which includes *Toxoplasma gondii*, avian influenza virus (specifically H5N1), African swine fever virus, molecular and genetic studies of animal and zoonotic pathogens, SARS-CoV-2, pseudorabies virus, microRNAs, and porcine circovirus.

The outline derived from this bibliometric analysis highlights the key implications for the research and strategic directions for CAAS and its VRIs. The upward trend that is observed in the NP accentuates its presence as a globally recognized contributor to veterinary research. The sustained research activity of CAAS showcases its commitment to maintaining and improving animal health and food security. The response to the swine fever viruses, as well as SARS-CoV-2, indicates the ability of these institutes to meet new threats that exist to the livestock sector in the present world. These findings indicate that CAAS should be supported by continuous investment, as it is important to sustain its efforts in disease prevention and to further improve the livestock sector. We observed that LVRI focused on parasitology and genomics, while SHVRI and HVRI focused on virology, highlighting CAAS’s diversified approaches. These approaches not only expand their research portfolio but also create opportunities for further research. For example, the combined integration of molecular genetic tools to enhance diagnostics, vaccines, and therapeutics. Policymakers and stakeholders can use these diverse approaches to optimize the implementation of management strategies and disease surveillance. The study also highlights the high-impact publication venues, such as Veterinary Microbiology and Parasites and Vectors, which served as the primary platforms for disseminating CAAS’s research. The strategic map presents implications such as the growing reliance on molecular and immunological approaches, which is evident due to the transition of “prrsv”, and “swine fever virus” from the center of the map to motor or niche themes in SHVRI. Moreover, ongoing research on viral diseases of swine and poultry is likely to remain a high priority, as there is a need to mitigate potential health or economic threats they may pose. Focus on *Toxoplasma gondii* has placed a special emphasis as a potential threat among parasitic diseases. Subsequent research on the interactions between hosts and parasites, and other aspects of parasitic diseases, is anticipated. Additionally, recent developments in molecular biology appear to influence future research directions for the VRIs of the CAAS. This bibliometric analysis may capture the research trends from 2009 to 2023. Several limitations must be acknowledged, which include the limited timeframe that may not capture long-term research trends or themes that were present before the 20th century and have emerged recently. Since VOSviewer is unable to differentiate between distinct authors having the same name, this limitation was addressed in this analysis and resolved. Restriction of the study to English-language documents in Scopus may overlook significant regional publications in Chinese. The limited time-frame of 2009–2023 is a constraint because citations continue to accumulate over time, the true total impact of the study published in the most recent years (e.g., 2022 and 2023) cannot be fairly compared against the works from 2013 without acknowledging this accumulation bias. Future directions may involve further research into emerging diseases such as African swine fever, SARS-CoV-2, and avian influenza with a special focus on genetic variations, transmission dynamics, and host-pathogen interactions so that their threat can be mitigated timely manner. Future research should investigate antimicrobial resistance in pathogens of zoonotic importance, which may primarily affect livestock, to inform policies. This aspect was not observed as a key research focus in CAAS. To further enhance the real-world applications of CAAS’s findings, further collaboration with industry stakeholders can enhance the practical implications of research outcomes. Furthermore, the strong collaborative framework, as demonstrated by co-authorship and coupling analyses, fosters diverse innovative solutions to complex problems in addition to having good relations. This model can act as an inspiration to national and international bodies for achieving interdisciplinary excellence.

## Conclusions

The study uses a bibliometric approach to assess the contributions of distinct VRIs of CAAS. The research questions outlined in the introduction are addressed using a range of analytical approaches. The analysis revealed that SHVRI, HVRI, and LVRI have significantly increased their publication output over the past few years. Moreover, the analysis demonstrated each institute’s distinct identity. SHVRI provided the foundational technical standards in virology, HVRI served as the high-impact center for emerging zoonotic threats, and lastly, LVRI produced the highest volume of research articles for parasitology and genomics. The co-authorship analysis demonstrated strong national and international collaborations, as well as social networks within the institute, represented as focus groups, paving the way for exciting research prospects. The study highlights the academy’s role in rapidly responding to the threat of African swine fever following its emergence in 2018. However, a strategic void was observed in the research of antimicrobial resistance of zoonotic pathogens as a result of mapping the research clusters. Core themes were identified through bibliographic coupling, revealing the major research foci of each institute. These include Newcastle disease virus, *Schistosoma japonicum*, African swine fever virus, Avian influenza, *Toxoplasma gondii*, etc. Veterinary research has benefited immensely from the VRIs of the CAAS, particularly in virology, parasitology, and immunology. Given LVRI’s established and growing experience in whole-genome sequencing of parasitic pathogens, as shown by the prevalence of genomics-focused clusters in its keyword co-occurrence maps, future research investments should strategically expand this capability to proactive surveillance of zoonotic spillover events, especially at the interface of wildlife and livestock, where African swine fever and new influenza strains have historically been seen to appear. This is an evolution of the current strengths of the institution, evidence-based and logical, but not a complete shift of the focus. Furthermore, taking into account the academy’s mandate to protect national food security, as well as in the proactive reaction to emerging challenges such as African swine fever, it becomes obligatory that future funding frameworks formally integrate industry-related cooperation. This would ensure that high-end specialized genomic and immunological research is effectively converted into commercially feasible diagnostic assays and vaccine candidates, which would elevate the scale of the “niche” scientific excellence into a practical biosecurity implementation effort. 

## Supplementary Information

Supplementary Material 1.

## Data Availability

All the data supporting this article is available from the corresponding author upon request.
