# Supplementary material for: Collaboration, impact, and research trends at the veterinary research institutes of the Chinese Academy of Agricultural Sciences: a bibliometric analysis from 2009 to 2023
Source: BMC Vet Res. 2026 May 16;22:406. doi: 10.1186/s12917-026-05531-7 (PMC13348813; doi:10.1186/s12917-026-05531-7)
Supplement: Supplementary file 1 — Supplementary Material 1. [file 12917_2026_5531_MOESM1_ESM.docx]

**SUPPLEMENTARY FILE**

**Table S1**. Top 20 most-cited articles from the Shanghai Veterinary Research Institute of the Chinese Academy of Agricultural Sciences, 2009–2023.

| Rank | Title | Year | Source title | Cited by | Document Type |
| --- | --- | --- | --- | --- | --- |
|  | Guidelines for the use and interpretation of assays for monitoring autophagy (3rd edition) | 2016 | Autophagy | 4347 | Review |
|  | The C/EBP homologous protein (CHOP) transcription factor functions in endoplasmic reticulum stress-induced apoptosis and microbial infection | 2019 | Frontiers in Immunology | 665 | Review |
|  | Globally diverse Toxoplasma gondii isolates comprise six major clades originating from a small number of distinct ancestral lineages | 2012 | PNAS USA | 291 | Article |
|  | Circulating miRNAs: Roles in cancer diagnosis, prognosis and therapy | 2015 | Advanced Drug Delivery Reviews | 278 | Review |
|  | The fecal virome of pigs on a high-density farm | 2011 | Journal of Virology | 273 | Article |
|  | Toxoplasma gondii infection in humans in China | 2011 | Parasites and Vectors | 242 | Review |
|  | The Nedd8-activating enzyme inhibitor MLN4924 induces autophagy and apoptosis to suppress liver cancer cell growth | 2012 | Cancer Research | 198 | Article |
|  | An infectious disease of ducks caused by a newly emerged Tembusu virus strain in mainland China | 2011 | Virology | 193 | Article |
|  | Importation and recombination are responsible for the latest emergence of highly pathogenic porcine reproductive and respiratory syndrome virus in China | 2015 | Journal of Virology | 184 | Article |
|  | A genome-wide CRISPR screen identifies host factors that regulate SARS-CoV-2 entry | 2021 | Nature Communications | 169 | Article |
|  | Cordyceps militaris polysaccharides can enhance the immunity and antioxidation activity in immunosuppressed mice | 2012 | Carbohydrate Polymers | 151 | Article |
|  | Molecular characterization of S. japonicum exosome-like vesicles reveals their regulatory roles in parasite-host interactions | 2016 | Scientific Reports | 128 | Article |
|  | An attenuated live vaccine based on highly pathogenic porcine reproductive and respiratory syndrome virus (HP-PRRSV) protects piglets against HP-PRRS | 2009 | Veterinary Microbiology | 116 | Article |
|  | Homologous 2’,5’-phosphodiesterases from disparate RNA viruses antagonize antiviral innate immunity | 2013 | PNAS USA | 107 | Article |
|  | Graphene Oxides Decorated with Carnosine as an Adjuvant to Modulate Innate Immune and Improve Adaptive Immunity in Vivo | 2016 | ACS Nano | 102 | Article |
|  | Schistosoma japonicum extracellular vesicle mirna cargo regulates host macrophage functions facilitating parasitism | 2019 | PLoS Pathogens | 100 | Article |
|  | Outbreaks of serotype 4 fowl adenovirus with novel genotype, China | 2016 | Emerging Microbes and Infections | 99 | Letter |
|  | OmpA is a virulence factor of Riemerella anatipestifer | 2011 | Veterinary Microbiology | 98 | Article |
|  | Emergence of a Pseudorabies virus variant with increased virulence to piglets | 2015 | Veterinary Microbiology | 97 | Article |
|  | Genomic characterization of emergent pseudorabies virus in China reveals marked sequence divergence: Evidence for the existence of two major genotypes | 2015 | Virology | 96 | Article |

**Table S2**. Top 20 most-cited articles from the Harbin Veterinary Research Institute of the Chinese Academy of Agricultural Sciences, 2009–2023.

| Rank | Title | Year | Source title | Cited by | Document Type |
| --- | --- | --- | --- | --- | --- |
| 1 | Susceptibility of ferrets, cats, dogs, and other domesticated animals to SARS-coronavirus 2 | 2020 | Science | 1317 | Article |
| 2 | Omicron variant of SARS-CoV-2: Genomics, transmissibility, and responses to current COVID-19 vaccines | 2022 | Journal of Medical Virology | 555 | Review |
| 3 | COVID-19: Epidemiology, Evolution, and Cross-Disciplinary Perspectives | 2020 | Trends in Molecular Medicine | 424 | Review |
| 4 | Emergence of African Swine Fever in China, 2018 | 2018 | Transboundary and Emerging Diseases | 423 | Article |
| 5 | Honeysuckle-encoded atypical microRNA2911 directly targets influenza A viruses | 2015 | Cell Research | 364 | Article |
| 6 | H7N9 influenza viruses are transmissible in ferrets by respiratory droplet | 2013 | Science | 356 | Article |
| 7 | Role for migratory wild birds in the global spread of avian influenza H5N8 | 2016 | Science | 335 | Article |
| 8 | Identification of amino acids in HA and PB2 critical for the transmission of H5N1 avian influenza viruses in a mammalian host | 2009 | PLoS Pathogens | 334 | Article |
| 9 | The duck genome and transcriptome provide insight into an avian influenza virus reservoir species | 2013 | Nature Genetics | 279 | Article |
| 10 | Architecture of African swine fever virus and implications for viral assembly | 2019 | Science | 264 | Article |
| 11 | Human monoclonal antibodies block the binding of SARS-CoV-2 spike protein to angiotensin converting enzyme 2 receptor | 2020 | Cellular and Molecular Immunology | 263 | Letter |
| 12 | Replication and virulence in pigs of the first African swine fever virus isolated in China | 2019 | Emerging Microbes and Infections | 256 | Article |
| 13 | MDA5 Governs the Innate Immune Response to SARS-CoV-2 in Lung Epithelial Cells | 2021 | Cell Reports | 255 | Article |
| 14 | Review of Influenza A Virus in Swine Worldwide: A Call for Increased Surveillance and Research | 2014 | Zoonoses and Public Health | 224 | Review |
| 15 | Genetics, Receptor Binding Property, and Transmissibility in Mammals of Naturally Isolated H9N2 Avian Influenza Viruses | 2014 | PLoS Pathogens | 223 | Article |
| 16 | A seven-gene-deleted African swine fever virus is safe and effective as a live attenuated vaccine in pigs | 2020 | Science China Life Sciences | 214 | Article |
| 17 | A single dose of an adenovirus-vectored vaccine provides protection against SARS-CoV-2 challenge | 2020 | Nature Communications | 210 | Article |
| 18 | Two amino acid residues in the matrix protein M1 contribute to the virulence difference of H5N1 avian influenza viruses in mice | 2009 | Virology | 205 | Article |
| 19 | HIP1R targets PD-L1 to lysosomal degradation to alter T cell–mediated cytotoxicity | 2019 | Nature Chemical Biology | 201 | Article |
| 20 | H5N1 hybrid viruses bearing 2009/H1N1 virus genes transmit in guinea pigs by respiratory droplet | 2013 | Science | 197 | Article |

**Table S3.** Top 20 most-cited articles from the Lanzhou Veterinary Research Institute of the Chinese Academy of Agricultural Sciences, 2009–2023.

| **Rank** | **Title** | **Year** | **Source title** | **Cited by** | **Document Type** |
| --- | --- | --- | --- | --- | --- |
| 1 | Guidelines for the use and interpretation of assays for monitoring autophagy (4th edition)1 | 2021 | Autophagy | 1494 | Review |
| 2 | The genomes of four tapeworm species reveal adaptations to parasitism | 2013 | Nature | 557 | Article |
| 3 | Severe fever with thrombocytopenia syndrome, an emerging tick-borne zoonosis | 2014 | The Lancet Infectious Diseases | 405 | Review |
| 4 | Geographical patterns of Toxoplasma gondii genetic diversity revealed by multilocus PCR-RFLP genotyping | 2014 | Parasitology | 327 | Article |
| 5 | Diagnosis of toxoplasmosis and typing of Toxoplasma gondii | 2015 | Parasites and Vectors | 319 | Review |
| 6 | Enhanced proliferation and osteogenic differentiation of mesenchymal stem cells on graphene oxide-incorporated electrospun poly(lactic-co-glycolic acid) nanofibrous mats | 2015 | ACS Applied Materials and Interfaces | 295 | Article |
| 7 | Globally diverse Toxoplasma gondii isolates comprise six major clades originating from a small number of distinct ancestral lineages | 2012 | Proceedings of the National Academy of Sciences of the United States of America | 291 | Article |
| 8 | Toxoplasma gondii infection in humans in China | 2011 | Parasites and Vectors | 242 | Review |
| 9 | Toxoplasma gondii infection in immunocompromised patients: A systematic review and meta-analysis | 2017 | Frontiers in Microbiology | 229 | Article |
| 10 | Genetics, Receptor Binding Property, and Transmissibility in Mammals of Naturally Isolated H9N2 Avian Influenza Viruses | 2014 | PLoS Pathogens | 223 | Article |
| 11 | Local admixture of amplified and diversified secreted pathogenesis determinants shapes mosaic Toxoplasma gondii genomes | 2016 | Nature Communications | 196 | Article |
| 12 | The genome and developmental transcriptome of the strongylid nematode Haemonchus contortus | 2013 | Genome Biology | 186 | Article |
| 13 | Prevalence and burden of Toxoplasma gondii infection in HIV-infected people: a systematic review and meta-analysis | 2017 | The Lancet HIV | 182 | Article |
| 14 | The leader proteinase of foot-and-mouth disease virus negatively regulates the type I interferon pathway by acting as a viral deubiquitinase | 2011 | Journal of Virology | 164 | Article |
| 15 | Roles and applications of probiotic Lactobacillus strains | 2018 | Applied Microbiology and Biotechnology | 161 | Review |
| 16 | Molecular survey and genetic identification of Anaplasma Species in goats from central and southern China | 2012 | Applied and Environmental Microbiology | 159 | Article |
| 17 | Immunization with Toxoplasma gondii GRA17 deletion mutant induces partial protection and survival in challenged mice | 2017 | Frontiers in Immunology | 146 | Article |
| 18 | Exosome-mediated microRNA transfer plays a role in radiation-induced bystander effect | 2015 | RNA Biology | 143 | Article |
| 19 | Sensitive detection of Escherichia coli O157:H7 using Pt-Au bimetal nanoparticles with peroxidase-like amplification | 2016 | Biosensors and Bioelectronics | 142 | Article |
| 20 | Toxocariasis: A silent threat with a progressive public health impact | 2018 | Infectious Diseases of Poverty | 140 | Review |
| 20 | USP13 negatively regulates antiviral responses by deubiquitinating STING | 2017 | Nature Communications | 140 | Article |
